# Supplementary figures and images for: Effects of LED light spectra and intensity on winter citrus nursery production
Source: PLoS One. 2026 Apr 30;21(4):e0347764. doi: 10.1371/journal.pone.0347764 (PMC13132180; doi:10.1371/journal.pone.0347764)

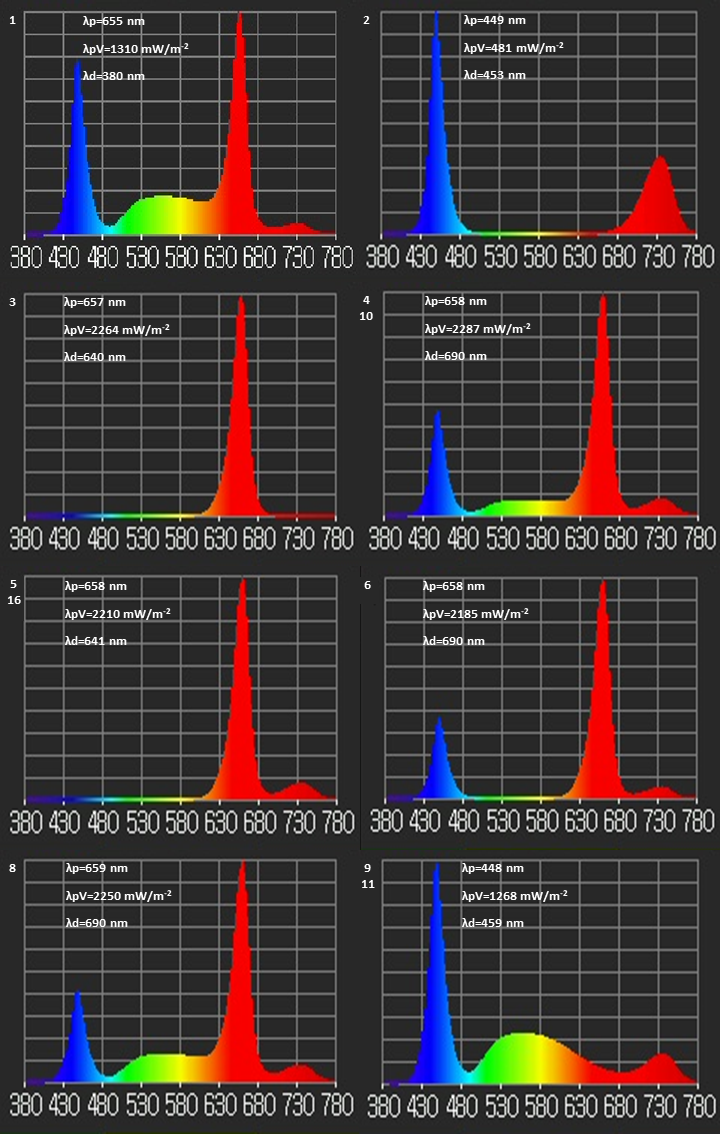

Supplement: S1 Fig — Runs 1–11. (TIF) [file pone.0347764.s001.tif]

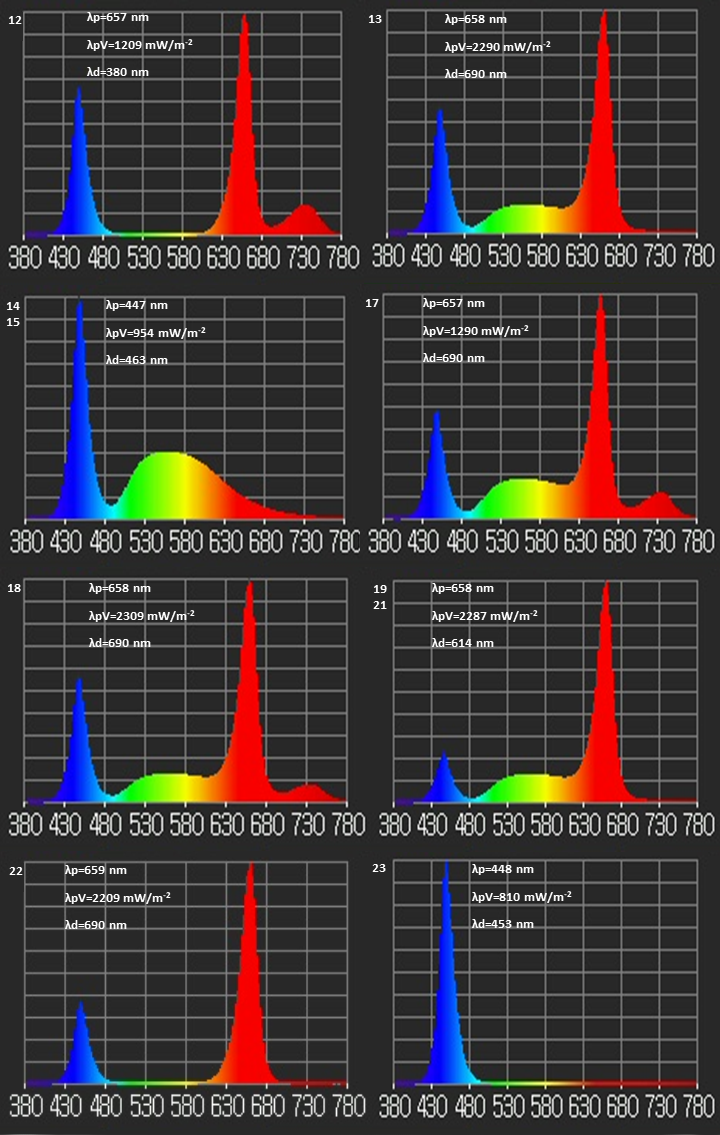

Supplement: S2 Fig — Runs 12–23. (TIF) [file pone.0347764.s002.tif]

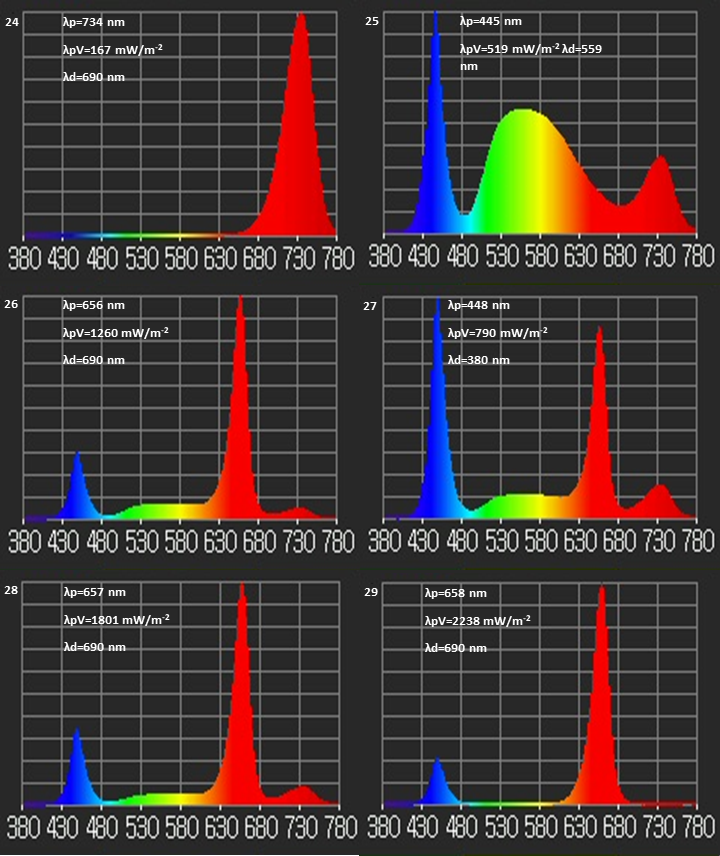

Supplement: S3 Fig — Runs 24–29. (TIF) [file pone.0347764.s003.tif]
