# Supplementary material for: Effects of LED light spectra and intensity on winter citrus nursery production
Source: PLoS One. 2026 Apr 30;21(4):e0347764. doi: 10.1371/journal.pone.0347764 (PMC13132180; doi:10.1371/journal.pone.0347764)
Supplement: S2 File — ANOVA tables and model equations in both coded and actual forms. Coded equations use factor ranges from −1 to +1, allowing easy comparison of effect sizes, while actual equations predict responses at specific factor settings. (DOCX) [file pone.0347764.s005.docx]

ANOVA tables and model equations in both coded and actual forms. Coded equations use factor ranges from -1 to +1, allowing easy comparison of effect sizes, while actual equations predict responses at specific factor settings.

Budbreak (%)

*Carrizo*

| **Source** | **Sum of Squares** | **df** | **Mean Square** | **F-value** | **p-value** |
| --- | --- | --- | --- | --- | --- |
| **Model** | 6343.27 | 5 | 1268.65 | 4.78 | 0.0005 |
| A-Red | 2529.44 | 1 | 2529.44 | 9.53 | 0.0025 |
| B-Blue | 1602.85 | 1 | 1602.85 | 6.04 | 0.0155 |
| D-Far-red | 44.84 | 1 | 44.84 | 0.1690 | 0.6818 |
| AD | 1587.59 | 1 | 1587.59 | 5.98 | 0.0160 |
| A² | 948.09 | 1 | 948.09 | 3.57 | 0.0613 |
| **Residual** | 30253.38 | 114 | 265.38 |  |  |
| Lack of Fit | 7926.99 | 17 | 466.29 | 2.03 | 0.0166 |
| Pure Error | 22326.39 | 97 | 230.17 |  |  |
| **Cor Total** | 36596.64 | 119 |  |  |  |

Percentage Budbreak (coded) = 43.87 - 5.19 * Red + 4.51 * Blue + 0.6907 * Far-red + 4.20 * Red * Far-red + 7.63 * Red²

Percentage Budbreak (actual) = 55.68 - 0.0493 * Red + 0.0090 * Blue - 0.0070 * Far-red + 1.70e-05 * Red * Far-red + 3.10e-05 * Red²

*Rich 16-6*

| **Source** | **Sum of Squares** | **df** | **Mean Square** | **F-value** | **p-value** |
| --- | --- | --- | --- | --- | --- |
| **Model** | 67.03 | 5 | 13.41 | 5.55 | 0.0001 |
| A-Red | 0.4324 | 1 | 0.4324 | 0.1790 | 0.6730 |
| B-Blue | 12.22 | 1 | 12.22 | 5.06 | 0.0264 |
| C-White | 24.58 | 1 | 24.58 | 10.18 | 0.0018 |
| D-Far-red | 1.06 | 1 | 1.06 | 0.4384 | 0.5092 |
| AD | 21.31 | 1 | 21.31 | 8.82 | 0.0036 |
| **Residual** | 275.34 | 114 | 2.42 |  |  |
| Lack of Fit | 35.67 | 17 | 2.10 | 0.8491 | 0.6336 |
| Pure Error | 239.68 | 97 | 2.47 |  |  |
| **Cor Total** | 342.37 | 119 |  |  |  |

Sqrt(Budbreak + 0.10) (coded) = 5.12 + 0.0676 * Red + 0.3998 * Blue + 0.5147 * White - 0.1062 * Far-red + 0.4875 * Red * Far-red

Sqrt(Budbreak + 0.10) (actual) = 4.73 – 8.40e-04 * Red + 8.00e-04 * Blue + 0.0010 * White - 0.0012 * Far-red + 1.95e-06 * Red * Far-red

Scion shoot growth

*Carrizo*

| **Source** | **Sum of Squares** | **df** | **Mean Square** | **F-value** | **p-value** |
| --- | --- | --- | --- | --- | --- |
| **Model** | 3.016E+05 | 7 | 43083.44 | 6.25 | 3.41236E-06 |
| A-Red | 58509.92 | 1 | 58509.92 | 8.49 | 0.0043 |
| B-Blue | 34933.29 | 1 | 34933.29 | 5.07 | 0.0263 |
| C-White | 51111.46 | 1 | 51111.46 | 7.41 | 0.0075 |
| D-Far-red | 3072.81 | 1 | 3072.81 | 0.4458 | 0.5057 |
| AD | 27606.44 | 1 | 27606.44 | 4.00 | 0.0478 |
| BC | 75737.27 | 1 | 75737.27 | 10.99 | 0.0012 |
| BD | 17501.65 | 1 | 17501.65 | 2.54 | 0.1139 |
| **Residual** | 7.721E+05 | 112 | 6893.40 |  |  |
| Lack of Fit | 2.499E+05 | 15 | 16660.85 | 3.10 | 0.0004 |
| Pure Error | 5.221E+05 | 97 | 5382.97 |  |  |
| **Cor Total** | 1.074E+06 | 119 |  |  |  |

Scion shoot growth (coded) = 281.51 + 24.98 * Red + 21.83 * Blue + 24.06 * White + 5.88 * Far-red + 17.89 * Red * Far-red + 34.95 * Blue * White – 16.17 * Blue * Far-red

Scion shoot growth (actual) = 241.43 + 0.0142 * Red + 0.0061 * Blue - 0.0218 * White + 0.0083 * Far-red + 7.16e-05 * Red * Far-red + 1.49e-04 * Blue * White - 6.47e-05 * Blue * Far-red

*Rich 16-6*

| **Source** | **Sum of Squares** | **df** | **Mean Square** | **F-value** | **p-value** |
| --- | --- | --- | --- | --- | --- |
| **Model** | 96333.65 | 6 | 16055.61 | 3.85 | 0.0016 |
| A-Red | 7357.64 | 1 | 7357.64 | 1.76 | 0.1870 |
| B-Blue | 2752.12 | 1 | 2752.12 | 0.6594 | 0.4185 |
| C-White | 14611.58 | 1 | 14611.58 | 3.50 | 0.0640 |
| D-Far-red | 1919.99 | 1 | 1919.99 | 0.4600 | 0.4990 |
| AD | 20711.16 | 1 | 20711.16 | 4.96 | 0.0280 |
| B² | 35703.36 | 1 | 35703.36 | 8.55 | 0.0042 |
| **Residual** | 4.549E+05 | 109 | 4173.50 |  |  |
| Lack of Fit | 65336.32 | 16 | 4083.52 | 0.9748 | 0.4902 |
| Pure Error | 3.896E+05 | 93 | 4188.98 |  |  |
| **Cor Total** | 5.512E+05 | 115 |  |  |  |

Scion shoot growth (coded) = 149.16 + 9.07 * Red + 6.15 * Blue + 12.89 * White + 4.67 * Far-red + 15.63 * Red * Far-red + 42.46 * Blue²

Scion shoot growth (actual) = 174.47 - 0.0131 * Red - 0.1575 * Blue + 0.0258 * White - 0.02193 * Far-red + 6.25e-05 Red * Far-red + 1.70e-04 * Blue²

Scion shoot diameter

*Carrizo*

| **Source** | **Sum of Squares** | **df** | **Mean Square** | **F-value** | **p-value** |
| --- | --- | --- | --- | --- | --- |
| **Model** | 14.01 | 6 | 2.33 | 5.41 | 6.029E-05 |
| A-Red | 3.06 | 1 | 3.06 | 7.09 | 0.0089 |
| B-Blue | 0.8904 | 1 | 0.8904 | 2.06 | 0.1535 |
| C-White | 2.04 | 1 | 2.04 | 4.74 | 0.0316 |
| D-Far-red | 0.0353 | 1 | 0.0353 | 0.0818 | 0.7755 |
| AD | 1.92 | 1 | 1.92 | 4.46 | 0.0369 |
| BC | 3.35 | 1 | 3.35 | 7.77 | 0.0062 |
| **Residual** | 48.30 | 112 | 0.4312 |  |  |
| Lack of Fit | 13.27 | 16 | 0.8294 | 2.27 | 0.0074 |
| Pure Error | 35.03 | 96 | 0.3649 |  |  |
| **Cor Total** | 62.31 | 118 |  |  |  |

Scion shoot diameter (coded) = 3.52 + 0.1812 * Red + 0.1108 * Blue + 0.1499 * White + 0.0199 * Far-red + 0.1493 + Red * Far-red + 0.2316 * Blue * White

Scion shoot diameter (actual) = 3.4415 + 6.39e-05 * Red – 2.42e-04 * Blue – 1.63e-04 * White – 2.59e-04 * Far-red + 5.97e-07 + Red * Far-red + 9.27e-07 * Blue * White

*Rich 16-6*

| **Source** | **Sum of Squares** | **df** | **Mean Square** | **F-value** | **p-value** |
| --- | --- | --- | --- | --- | --- |
| **Model** | 9.89 | 6 | 1.65 | 4.88 | 0.0002 |
| A-Red | 3.87 | 1 | 3.87 | 11.47 | 0.0010 |
| B-Blue | 0.0061 | 1 | 0.0061 | 0.0181 | 0.8934 |
| C-White | 0.0042 | 1 | 0.0042 | 0.0123 | 0.9117 |
| AB | 0.9320 | 1 | 0.9320 | 2.76 | 0.0996 |
| AC | 0.7005 | 1 | 0.7005 | 2.07 | 0.1527 |
| B² | 1.61 | 1 | 1.61 | 4.78 | 0.0310 |
| **Residual** | 35.11 | 104 | 0.3376 |  |  |
| Lack of Fit | 3.73 | 16 | 0.2328 | 0.6529 | 0.8313 |
| Pure Error | 31.38 | 88 | 0.3566 |  |  |
| **Cor Total** | 45.00 | 110 |  |  |  |

Scion shoot diameter (coded) = 2.26 + 0.2132 * Red - 0.0094 * Blue - 0.0070 * White + 0.1232 * Red * Blue + 0.0971 * Red * White + 0.2906 * Blue²

Scion shoot diameter (actual) = 2.57 - 1.44e-05 * Red - 0.0014 * Blue - 0.0002 * White + 4.93e-07 * Red * Blue + 3.89e-07 * Red * White + 1.1623e-06 * Blue²

Rootstock diameter

*Carrizo*

| **Source** | **Sum of Squares** | **df** | **Mean Square** | **F-value** | **p-value** |
| --- | --- | --- | --- | --- | --- |
| **Model** | 11.86 | 7 | 1.69 | 14.32 | 3.49214E-13 |
| A-Red | 2.29 | 1 | 2.29 | 19.32 | 2.52781E-05 |
| B-Blue | 1.90 | 1 | 1.90 | 16.08 | 0.0001 |
| C-White | 3.79 | 1 | 3.79 | 32.05 | 1.17238E-07 |
| D-Far-red | 0.7298 | 1 | 0.7298 | 6.17 | 0.0145 |
| AB | 0.3005 | 1 | 0.3005 | 2.54 | 0.1138 |
| BC | 0.2934 | 1 | 0.2934 | 2.48 | 0.1182 |
| A² | 0.5494 | 1 | 0.5494 | 4.64 | 0.0333 |
| **Residual** | 13.25 | 112 | 0.1183 |  |  |
| Lack of Fit | 2.50 | 15 | 0.1668 | 1.51 | 0.1182 |
| Pure Error | 10.75 | 97 | 0.1108 |  |  |
| **Cor Total** | 25.11 | 119 |  |  |  |

Rootstock diameter (coded) = 7.03 + 0.1565 * Red + 0.1645 * Blue + 0.2039 * White – 0.0907 * Far-red + 0.0658 * Red * Blue + 0.0685 * Blue * White + 0.1900 * Red²

Rootstock diameter (actual) = 6.9172 – 5.79e-04 * Red + 6.06e-05 * Blue + 2.71e-04 * White – 1.81e-04 * Far-red + 2.63e-07 * Red * Blue + 2.74e-07 * Blue * White + 7.60e-07 * Red²

*Rich 16-6*

| **Source** | **Sum of Squares** | **df** | **Mean Square** | **F-value** | **p-value** |
| --- | --- | --- | --- | --- | --- |
| **Model** | 4.36 | 4 | 1.09 | 6.41 | 0.0001 |
| A-Red | 2.85 | 1 | 2.85 | 16.76 | 8.28782E-05 |
| C-White | 0.7019 | 1 | 0.7019 | 4.12 | 0.0448 |
| D-Far-red | 0.1313 | 1 | 0.1313 | 0.7712 | 0.3818 |
| CD | 0.6139 | 1 | 0.6139 | 3.61 | 0.0603 |
| **Residual** | 18.05 | 106 | 0.1703 |  |  |
| Lack of Fit | 2.84 | 18 | 0.1578 | 0.9131 | 0.5650 |
| Pure Error | 15.21 | 88 | 0.1728 |  |  |
| **Cor Total** | 22.41 | 110 |  |  |  |

Rootstock diameter (coded) = 5.39 + 0.1809 * Red + 0.0890 * White + 0.0382 * Far-red + 0.0875 * White * Far-red

Rootstock diameter (actual) = 5.17 + 3.6176 * Red + 2.93e-06 * White - 9.87e-05 * Far-red + 3.51e-07 * White * Far-red

Internode length

*Carrizo*

| **Source** | **Sum of Squares** | **df** | **Mean Square** | **F-value** | **p-value** |
| --- | --- | --- | --- | --- | --- |
| **Model** | 11864.20 | 6 | 1977.37 | 4.91 | 0.0002 |
| A-Red | 1636.00 | 1 | 1636.00 | 4.06 | 0.0462 |
| B-Blue | 1688.46 | 1 | 1688.46 | 4.19 | 0.0429 |
| C-White | 43.62 | 1 | 43.62 | 0.1083 | 0.7427 |
| D-Far-red | 438.82 | 1 | 438.82 | 1.09 | 0.2988 |
| AD | 1269.28 | 1 | 1269.28 | 3.15 | 0.0786 |
| BC | 3882.07 | 1 | 3882.07 | 9.64 | 0.0024 |
| **Residual** | 45515.95 | 113 | 402.80 |  |  |
| Lack of Fit | 12683.23 | 16 | 792.70 | 2.34 | 0.0056 |
| Pure Error | 32832.72 | 97 | 338.48 |  |  |
| **Cor Total** | 57380.15 | 119 |  |  |  |

(Internode length + 0.02)^1.49^ (coded) = 63.24 + 4.17 * Red + 4.77 * Blue + 0.6860 * White + 2.22 * Far-red + 3.83 * Red * Far-red + 7.83 * Blue * White

(Internodes + 0.02)^1.49^ (actual) = 63.05 + 6.81e-04 * Red - 0.0061 * Blue - 0.0143 * White - 0.0032 * Far-red + 1.53e-05 * Red * Far-red + 3.136e-05 * Blue * White

*Rich 16-6*

| **Source** | **Sum of Squares** | **df** | **Mean Square** | **F-value** | **p-value** |
| --- | --- | --- | --- | --- | --- |
| **Model** | 310.38 | 8 | 38.80 | 3.13 | 0.0034 |
| A-Red | 56.66 | 1 | 56.66 | 4.57 | 0.0350 |
| B-Blue | 0.1667 | 1 | 0.1667 | 0.0134 | 0.9080 |
| C-White | 6.68 | 1 | 6.68 | 0.5383 | 0.4648 |
| D-Far-red | 18.18 | 1 | 18.18 | 1.47 | 0.2289 |
| AC | 77.28 | 1 | 77.28 | 6.23 | 0.0142 |
| BC | 22.72 | 1 | 22.72 | 1.83 | 0.1791 |
| BD | 30.84 | 1 | 30.84 | 2.48 | 0.1180 |
| B² | 52.72 | 1 | 52.72 | 4.25 | 0.0418 |
| **Residual** | 1265.93 | 102 | 12.41 |  |  |
| Lack of Fit | 206.36 | 14 | 14.74 | 1.22 | 0.2727 |
| Pure Error | 1059.56 | 88 | 12.04 |  |  |
| **Cor Total** | 1576.31 | 110 |  |  |  |

Internode length (coded) = 11.4422 + 0.6869 + A - 0.1250 * B - 0.3746 * C + 0.4919 * D + 0.8930 * AC - 0.7031 * BD + 1.7187 * B²

Internode length (actual) = 13.00 – 3.78e-04 * Red - 0.0065 * Blue - 0.0038 * White + 0.0024 * Far-red + 4.03e-06 * Red * White + 2.47e-06 * Blue * White - 2.89e-06 * Blue * Far-red + 6.82e-06 * Blue²

Leaf area

*Carrizo*

| **Source** | **Sum of Squares** | **df** | **Mean Square** | **F-value** | **p-value** |
| --- | --- | --- | --- | --- | --- |
| **Model** | 1143.00 | 7 | 163.29 | 9.52 | 3.08543E-09 |
| A-Red | 129.36 | 1 | 129.36 | 7.54 | 0.0070 |
| B-Blue | 162.74 | 1 | 162.74 | 9.49 | 0.0026 |
| C-White | 171.87 | 1 | 171.87 | 10.02 | 0.0020 |
| D-Far-red | 0.0726 | 1 | 0.0726 | 0.0042 | 0.9482 |
| AD | 175.97 | 1 | 175.97 | 10.26 | 0.0018 |
| BC | 272.87 | 1 | 272.87 | 15.91 | 0.0001 |
| CD | 57.86 | 1 | 57.86 | 3.37 | 0.0689 |
| **Residual** | 1921.03 | 112 | 17.15 |  |  |
| Lack of Fit | 652.93 | 15 | 43.53 | 3.33 | 0.0002 |
| Pure Error | 1268.10 | 97 | 13.07 |  |  |
| **Cor Total** | 3064.03 | 119 |  |  |  |

Sqrt(Leaf area) (coded) = 20.53 + 1.18 * Red + 1.53 * Blue + 1.36 * White + 0.0286 * Far-red + 1.43 * Red * Far-red + 2.11 * Blue * White – 0.8451 * White * Far-red

Sqrt(Leaf area) (actual) = 19.12 – 5.00e-04 * Red - 0.0012 * Blue + 1.9917e-04 * White - 0.0011 * Far-red + 5.70e-06 * Red * Far-red + 8.43e-06 * Blue * White - 3.38e-06 * White * Far-red

*Rich 16-6*

| **Source** | **Sum of Squares** | **df** | **Mean Square** | **F-value** | **p-value** |
| --- | --- | --- | --- | --- | --- |
| **Model** | 230.34 | 7 | 32.91 | 4.57 | 0.0002 |
| A-Red | 36.66 | 1 | 36.66 | 5.09 | 0.0262 |
| B-Blue | 0.0462 | 1 | 0.0462 | 0.0064 | 0.9363 |
| C-White | 35.99 | 1 | 35.99 | 5.00 | 0.0276 |
| D-Far-red | 0.0591 | 1 | 0.0591 | 0.0082 | 0.9280 |
| AD | 41.11 | 1 | 41.11 | 5.71 | 0.0187 |
| BC | 17.89 | 1 | 17.89 | 2.48 | 0.1181 |
| B² | 68.52 | 1 | 68.52 | 9.51 | 0.0026 |
| **Residual** | 741.92 | 103 | 7.20 |  |  |
| Lack of Fit | 70.08 | 15 | 4.67 | 0.6119 | 0.8579 |
| Pure Error | 671.85 | 88 | 7.63 |  |  |
| **Cor Total** | 972.26 | 110 |  |  |  |

Sqrt(Leaf area) (coded) = 12.74 + 0.6599 * Red + 0.0261 * Blue + 0.6596 * White - 0.0270 * Far-red + 0.7296 * Red * Far-red + 0.5535 * Blue * White + 1.93 * Blue²

Sqrt(Leaf area) (actual) = 14.64 – 1.39e-04 * Red - 0.0088 * Blue + 0.0002 * White - 0.0015 * Far-red + 2.92e-06 * Red * Far-red + 2.21e-06 * Blue * White + 7.73e-06 * Blue²

Scion dry weight

*Carrizo*

| **Source** | **Sum of Squares** | **df** | **Mean Square** | **F-value** | **p-value** |
| --- | --- | --- | --- | --- | --- |
| **Model** | 10.96 | 6 | 1.83 | 10.21 | 5.2342E-09 |
| A-Red | 3.53 | 1 | 3.53 | 19.72 | 2.09994E-05 |
| B-Blue | 1.20 | 1 | 1.20 | 6.70 | 0.0109 |
| C-White | 1.67 | 1 | 1.67 | 9.34 | 0.0028 |
| D-Far-red | 0.0002 | 1 | 0.0002 | 0.0010 | 0.9753 |
| AD | 0.7970 | 1 | 0.7970 | 4.46 | 0.0370 |
| BC | 2.08 | 1 | 2.08 | 11.64 | 0.0009 |
| **Residual** | 20.21 | 113 | 0.1789 |  |  |
| Lack of Fit | 5.26 | 16 | 0.3286 | 2.13 | 0.0125 |
| Pure Error | 14.96 | 97 | 0.1542 |  |  |
| **Cor Total** | 31.17 | 119 |  |  |  |

Sqrt(Scion dry weight) (coded) = 2.23 + 1.936 * Red + 0.1272 * Blue + 0.1343 * White + 0.0014 * Far-red + 0.0959 * Red * Far-red + 0.1813 * Blue * White

Sqrt(Scion dry weight) (actual) = 2.05 + 1.95e-04 * Red – 1.08e-04 * Blue - 9.40e-05 * White – 1.8911 * Far-red + 3.84e-07 * Red * Far-red + 7.25e-07 * Blue * White

*Rich 16-6*

| **Source** | **Sum of Squares** | **df** | **Mean Square** | **F-value** | **p-value** |
| --- | --- | --- | --- | --- | --- |
| **Model** | 45.91 | 6 | 7.65 | 8.29 | 2.43477E-07 |
| A-Red | 17.25 | 1 | 17.25 | 18.68 | 3.55252E-05 |
| B-Blue | 0.0010 | 1 | 0.0010 | 0.0010 | 0.9744 |
| C-White | 7.58 | 1 | 7.58 | 8.20 | 0.0051 |
| D-Far-red | 3.97 | 1 | 3.97 | 4.29 | 0.0407 |
| AC | 2.63 | 1 | 2.63 | 2.85 | 0.0946 |
| B² | 4.43 | 1 | 4.43 | 4.79 | 0.0308 |
| **Residual** | 96.05 | 104 | 0.9235 |  |  |
| Lack of Fit | 17.52 | 16 | 1.10 | 1.23 | 0.2641 |
| Pure Error | 78.53 | 88 | 0.8923 |  |  |
| **Cor Total** | 141.96 | 110 |  |  |  |

Scion dry weight (coded) = 2.44 + 0.4504 * Red + 0.0037 * Blue + 0.3003 * White + 0.2172 * Far-red + 0.1828 * Red * White + 0.2218 * Blue²

Scion dry weight (actual) = 2.13 + 5.35e-04 * Red - 0.0019 * Blue + 2.35e-04 * White + 4.34ee-04 * Far-red + 7.31e-07 * Red * White + 1.94e-06 Blue²

Chlorophyll index

*Carrizo*

| **Source** | **Sum of Squares** | **df** | **Mean Square** | **F-value** | **p-value** |
| --- | --- | --- | --- | --- | --- |
| **Model** | 642.44 | 9 | 71.38 | 41.51 | 2.14206E-31 |
| A-Red | 9.69 | 1 | 9.69 | 5.63 | 0.0193 |
| B-Blue | 112.98 | 1 | 112.98 | 65.70 | 7.96558E-13 |
| C-White | 143.16 | 1 | 143.16 | 83.26 | 3.95628E-15 |
| D-Far-red | 260.52 | 1 | 260.52 | 151.51 | 2.04569E-22 |
| AB | 7.64 | 1 | 7.64 | 4.44 | 0.0373 |
| AD | 33.70 | 1 | 33.70 | 19.60 | 2.25995E-05 |
| BD | 5.94 | 1 | 5.94 | 3.46 | 0.0657 |
| CD | 25.43 | 1 | 25.43 | 14.79 | 0.0002 |
| C² | 78.32 | 1 | 78.32 | 45.55 | 7.28014E-10 |
| **Residual** | 189.14 | 110 | 1.72 |  |  |
| Lack of Fit | 135.30 | 13 | 10.41 | 18.75 | 5.2788E-21 |
| Pure Error | 53.85 | 97 | 0.5551 |  |  |
| **Cor Total** | 831.59 | 119 |  |  |  |

Chlorophyll index (coded) = 74.74 + 0.3236 * Red + 1.28 * Blue + 1.28 * White – 1.68 * Far-red + 0.3407 * Red * Blue + 0.6326 * Red * Far-red + 0.3062 * Blue * Far-red – 0.5781 * White * Far-red + 2.45 * White²

Chlorophyll index (actual) = 76.68 - 0.0013 * Red + 0.0013 * Blue - 0.0061 * White - 0.0041 * Far-red + 1.36e-06 * Red * Blue + 2.53e-06 * Red * Far-red + 1.22e-06 * Blue * Far-red - 2.31e-06 * White * Far-red + 9.80e-06 * White²

*Rich 16-6*

| **Source** | **Sum of Squares** | **df** | **Mean Square** | **F-value** | **p-value** |
| --- | --- | --- | --- | --- | --- |
| **Model** | 547.60 | 8 | 68.45 | 18.84 | 1.33732E-17 |
| A-Red | 74.58 | 1 | 74.58 | 20.52 | 1.49139E-05 |
| B-Blue | 270.33 | 1 | 270.33 | 74.39 | 5.09601E-14 |
| C-White | 18.19 | 1 | 18.19 | 5.01 | 0.0273 |
| D-Far-red | 56.49 | 1 | 56.49 | 15.55 | 0.0001 |
| BD | 19.15 | 1 | 19.15 | 5.27 | 0.0236 |
| CD | 20.55 | 1 | 20.55 | 5.65 | 0.0191 |
| A² | 42.17 | 1 | 42.17 | 11.60 | 0.0009 |
| B² | 19.98 | 1 | 19.98 | 5.50 | 0.0208 |
| **Residual** | 403.35 | 111 | 3.63 |  |  |
| Lack of Fit | 240.22 | 14 | 17.16 | 10.20 | 1.13751E-13 |
| Pure Error | 163.13 | 97 | 1.68 |  |  |
| **Cor Total** | 950.95 | 119 |  |  |  |

Chlorophyll index (coded) = 78.17 + 0.9088 * Red + 1.94 * Blue + 0.4663 * White - 0.7826 * Far-red + 0.5508 * Blue * Far-red – 0.5180 * White * Far-red + 1.81 * Red² - 1.10 * Blue²

Chlorophyll index (actual) = 76.38 - 0.0054 * Red + 0.0072 * Blue + 0.0020 * White - 0.0016 * Far-red + 2.20e-06 * Blue * Far-red -2.07e-06 * White * Far-red + 7.23e-06 * Red² - 4.40e-06 * Blue²
