# Supplementary material for: Effects of LED light spectra and intensity on winter citrus nursery production
Source: PLoS One. 2026 Apr 30;21(4):e0347764. doi: 10.1371/journal.pone.0347764 (PMC13132180; doi:10.1371/journal.pone.0347764)
Supplement: Attachment — Submitted filename: PONE-D-25-45421.pdf [file pone.0347764.s006.pdf]

PLOS ONE

Effects of LED light spectra and intensity on winter citrus nursery production

--Manuscript Draft--

**Manuscript Number:**	PONE-D-25-45421
**Article Type:**	Research Article
**Full Title:**	Effects of LED light spectra and intensity on winter citrus nursery production
**Short Title:**	Citrus Growth Under LEDs
**Corresponding Author:**	Randall P. Niedz<br>US Department of Agriculture<br>Ft. Pierce, Florida UNITED STATES OF AMERICA
**Keywords:**	sweet orange; Citrus sinensis; Poncirus trifoliata; Washington Navel; Carrizo; Rich 16-6; grafting; plant propagation; bud growth; budbreak; response surface methodology; RSM, design of experiments; DOE; rootstock
**Abstract:**	This study investigated the effects of supplemental light-emitting diode (LED) light spectra and intensity on the winter growth of grafted citrus nursery plants. The primary objective was to determine how different combinations of red, blue, white, and far-red LED light, applied at varying intensities, influence vegetative budbreak and scion shoot growth in young citrus trees during short winter days.<br>The experiment used two citrus rootstocks, Carrizo (a hybrid of sweet orange and trifoliate orange) and Rich 16-6 (trifoliate orange), each budded with Washington Navel orange. Plants were grown in a temperature-controlled greenhouse and received natural sunlight, with supplemental LED lighting used to extend the photoperiod to 16 hours. The study employed a 4-factor response surface design, testing multiple combinations of light spectra and intensity. Eight plant growth and physiological responses were measured, including percentage budbreak, scion shoot length and diameter, rootstock diameter, internode length, leaf area, scion dry weight, and leaf chlorophyll index.<br>Results showed that both the quality and intensity of supplemental LED light significantly affected all measured responses. Blue and white light, particularly at higher intensities, increased vegetative budbreak in both rootstocks, while red and far-red light reduced budbreak. The greatest scion shoot growth was observed when red, blue, and white light were combined at maximum intensity. Far-red light alone did not enhance growth, but when combined with red or white light, it further increased shoot elongation and leaf area. Chlorophyll content was highest under blue and white light and lowest under far-red light.<br>These findings demonstrate that optimizing supplemental LED light spectra and intensity can improve winter nursery production of grafted citrus by enhancing budbreak and shoot growth. The results provide a basis for refining light management strategies in commercial citrus nurseries to increase propagation efficiency during periods of limited natural light.
**Order of Authors:**	Randall P. Niedz
	Rayane Bisi
	Ute Albrecht
	Kim Bowman
**Additional Information:**	
**Question**	**Response**
**Financial Disclosure**<br><br>Enter a financial disclosure statement that describes the sources of funding for the work included in this submission. Review the submission guidelines for detailed	Yes

Powered by Editorial Manager® and ProduXion Manager® from Aries Systems Corporation

requirements. View published research articles from *PLOS ONE* for specific examples.<br><br>This statement is required for submission and **will appear in the published article** if the submission is accepted. Please make sure it is accurate.<br><br>**Funded studies**<br>Enter a statement with the following details:<br>• Initials of the authors who received each award<br>• Grant numbers awarded to each author<br>• The full name of each funder<br>• URL of each funder website<br>• Did the sponsors or funders play any role in the study design, data collection and analysis, decision to publish, or preparation of the manuscript?<br><br>Did you receive funding for this work?	
Please add funding details.<br>as follow-up to "**Financial Disclosure**<br><br>Enter a financial disclosure statement that describes the sources of funding for the work included in this submission. Review the submission guidelines for detailed requirements. View published research articles from *PLOS ONE* for specific examples.<br><br>This statement is required for submission and **will appear in the published article** if the submission is accepted. Please make sure it is accurate.	This research was conducted at the United States Horticultural Research Laboratory, Ft Pierce, Florida, USA and was funded by the California Citrus Nursery Board (ALB-21-23 and BOW-21-23).

**Funded studies**<br>Enter a statement with the following details:<br>• Initials of the authors who received each award<br>• Grant numbers awarded to each author<br>• The full name of each funder<br>• URL of each funder website<br>• Did the sponsors or funders play any role in the study design, data collection and analysis, decision to publish, or preparation of the manuscript?<br><br>Did you receive funding for this work?"	
Please select the country of your main research funder (please select carefully as in some cases this is used in fee calculation).<br>as follow-up to "**Financial Disclosure**<br><br>Enter a financial disclosure statement that describes the sources of funding for the work included in this submission. Review the submission guidelines for detailed requirements. View published research articles from *PLOS ONE* for specific examples.<br><br>This statement is required for submission and **will appear in the published article** if the submission is accepted. Please make sure it is accurate.<br><br>**Funded studies**<br>Enter a statement with the following details:<br>• Initials of the authors who received each award<br>• Grant numbers awarded to each author<br>• The full name of each funder<br>• URL of each funder website<br>• Did the sponsors or funders play any role in the study design, data collection and analysis, decision to publish, or preparation of the manuscript?<br><br>Did you receive funding for this work?"	UNITED STATES - US
**Competing Interests**	The authors have declared that no competing interests exist.

Use the instructions below to enter a competing interest statement for this submission. On behalf of all authors, disclose any competing interests that could be perceived to bias this work—acknowledging all financial support and any other relevant financial or non-financial competing interests.<br><br>This statement is required for submission and **will appear in the published article** if the submission is accepted. Please make sure it is accurate and that any funding sources listed in your Funding Information later in the submission form are also declared in your Financial Disclosure statement.<br><br>View published research articles from *PLOS ONE* for specific examples.<br><br>**NO authors have competing interests**<br><br>Enter: *The authors have declared that no competing interests exist.*<br><br>**Authors with competing interests**<br><br>Enter competing interest details beginning with this statement:<br><br>*I have read the journal's policy and the authors of this manuscript have the following competing interests: [insert competing interests here]*<br><br>* typeset	
**Ethics Statement**<br><br>Enter an ethics statement for this submission. This statement is required if the study involved:<br><br>• Human participants<br>• Human specimens or tissue<br>• Vertebrate animals or cephalopods<br>• Vertebrate embryos or tissues	N/A

• Field research<br><br>Write "N/A" if the submission does not require an ethics statement.<br><br>General guidance is provided below. Consult the submission guidelines for detailed instructions. **Make sure that all information entered here is included in the Methods section of the manuscript.**	

Powered by Editorial Manager® and ProduXion Manager® from Aries Systems Corporation

**Format for specific study types**<br><br>**Human Subject Research (involving human participants and/or tissue)**<br>• Give the name of the institutional review board or ethics committee that approved the study<br>• Include the approval number and/or a statement indicating approval of this research<br>• Indicate the form of consent obtained (written/oral) or the reason that consent was not obtained (e.g. the data were analyzed anonymously)<br><br>**Animal Research (involving vertebrate animals, embryos or tissues)**<br>• Provide the name of the Institutional Animal Care and Use Committee (IACUC) or other relevant ethics board that reviewed the study protocol, and indicate whether they approved this research or granted a formal waiver of ethical approval<br>• Include an approval number if one was obtained<br>• If the study involved *non-human primates*, add *additional details* about animal welfare and steps taken to ameliorate suffering<br>• If anesthesia, euthanasia, or any kind of animal sacrifice is part of the study, include briefly which substances and/or methods were applied<br><br>**Field Research**<br><br>Include the following details if this study involves the collection of plant, animal, or other materials from a natural setting:<br>• Field permit number<br>• Name of the institution or relevant body that granted permission	
**Data Availability**<br><br>Authors are required to make all data underlying the findings described fully available, without restriction, and from the time of publication. PLOS allows rare exceptions to address legal and ethical concerns. See the PLOS Data Policy and FAQ for detailed information.	Yes - all data are fully available without restriction

A Data Availability Statement describing where the data can be found is required at submission. Your answers to this question constitute the Data Availability Statement and **will be published in the article**, if accepted.<br><br>**Important:** Stating 'data available on request from the author' is not sufficient. If your data are only available upon request, select 'No' for the first question and explain your exceptional situation in the text box.<br><br>Do the authors confirm that all data underlying the findings described in their manuscript are fully available without restriction?	
**Describe where the data may be found in full sentences. If you are copying our sample text, replace any instances of XXX with the appropriate details.**<br><br>• If the data are **held or will be held in a public repository**, include URLs, accession numbers or DOIs. If this information will only be available after acceptance, indicate this by ticking the box below. For example: *All XXX files are available from the XXX database (accession number(s) XXX, XXX.*).<br>• If the data are all contained **within the manuscript and/or Supporting Information files**, enter the following: *All relevant data are within the manuscript and its Supporting Information files.*<br>• If neither of these applies but you are able to provide **details of access elsewhere**, with or without limitations, please do so. For example:<br><br>*Data cannot be shared publicly because of [XXX]. Data are available from the XXX Institutional Data Access / Ethics Committee (contact via XXX) for researchers who meet the criteria for access to confidential data.*<br><br>*The data underlying the results presented in the study are available from (include the name of the third party*	All relevant data are within the manuscript and its Supporting Information files.

and contact information or URL).<br>• This text is appropriate if the data are owned by a third party and authors do not have permission to share the data.<br><br>* typeset	
Additional data availability information:	

Powered by Editorial Manager® and ProduXion Manager® from Aries Systems Corporation

Manuscript Click here to access/download;Manuscript;MS_Bisi_LED_8-19-2025.docx

Effects of LED light spectra and intensity on winter citrus nursery production

Rayane Barcelos Bisi[1,2], Ute Albrecht[1], Randall P. Niedz[2*], Kim D. Bowman[2]

[1]Southwest Florida Research and Education Center, University of Florida/IFAS, Immokalee, Florida, United States of America

[2]U.S. Horticultural Research Laboratory, USDA, ARS, Fort Pierce, Florida, United States of America

[*] Corresponding author. Email: randall.niedz@usda.gov

Abstract

This study investigated the effects of supplemental light-emitting diode (LED) light spectra and intensity on the winter growth of grafted citrus nursery plants. The primary objective was to determine how different combinations of red, blue, white, and far-red LED light, applied at varying intensities, influence vegetative budbreak and scion shoot growth in young citrus trees during short winter days.

The experiment used two citrus rootstocks, Carrizo (a hybrid of sweet orange and trifoliate orange) and Rich 16-6 (trifoliate orange), each budded with Washington Navel orange. Plants were grown in a temperature-controlled greenhouse and received natural sunlight, with supplemental LED lighting used to extend the photoperiod to 16 hours. The study employed a 4-factor response surface design, testing multiple combinations of light spectra and intensity. Eight plant growth and physiological responses were measured, including percentage budbreak, scion shoot length and diameter, rootstock diameter, internode length, leaf area, scion dry weight, and leaf chlorophyll index.

Results showed that both the quality and intensity of supplemental LED light significantly affected all measured responses. Blue and white light, particularly at higher intensities, increased vegetative budbreak in both rootstocks, while red and far-red light reduced budbreak. The greatest scion shoot growth was observed when red, blue, and white light were combined at maximum intensity. Far-red light alone did not enhance growth, but when combined with red or white light, it further increased shoot elongation and leaf area. Chlorophyll content was highest under blue and white light and lowest under far-red light.

These findings demonstrate that optimizing supplemental LED light spectra and intensity can improve winter nursery production of grafted citrus by enhancing budbreak and shoot growth.

The results provide a basis for refining light management strategies in commercial citrus nurseries to increase propagation efficiency during periods of limited natural light.

Introduction

Citrus production in commercial nurseries relies on the propagation (grafting) of scion cultivars on selected rootstocks, a process that is sensitive to environmental conditions during the winter months. Reduced daylength and lower temperatures during this period limits vegetative budbreak and the subsequent growth of grafted scions, thereby extending the nursery production cycle and potentially affecting overall productivity. To address these limitations, supplemental lighting was employed to extend the daylength and improve plant growth during the winter, with several studies in citrus and other horticultural crops demonstrating positive effects on budbreak and shoot development [1-7].

Light is a fundamental environmental factor that regulates plant physiology throughout the life cycle, functioning as both an energy source for photosynthesis and a signal that controls a range of developmental processes [8]. The response of plants to light is complex and depends on several parameters, including intensity, photoperiod, and spectral composition [9-11]. Recent advances in LED technology have made it possible to manipulate light spectra with precision, enabling the study of specific wavelength effects-such as blue, red, and far-red light-on plant growth and development [12]. While photosynthetically active radiation (PAR) is traditionally defined as 400–700 nm, far-red light (700–800 nm) has a role in regulating phytochrome-mediated responses that influence plant morphology and development [9, 13-15].

In controlled environments, supplemental lighting is commonly used to achieve a target daily light integral (DLI), particularly in regions or seasons with limited natural sunlight. However, both insufficient and excessive light intensity can have adverse effects: low intensity may not support optimal growth, while high intensity can induce stress responses, including the production of antioxidants and non-photosynthetic pigments to mitigate photodamage [16-18].

Plants have evolved diverse photoprotection mechanisms to optimize photosynthesis and prevent photoinhibition under fluctuating light conditions.

The literature indicates that plant responses to supplemental light vary widely among species and even among cultivars within a species [19-21]. While the effects of light spectra and intensity have been extensively studied in various horticultural crops, there is little information in the citrus literature regarding the interactive effects of these factors on budbreak and early growth. This knowledge is essential for optimizing nursery production practices, particularly during the winter when natural light is limited.

The role of far-red light in plant growth is not completely understood. Although far-red photons are inefficient at driving photosynthesis when applied alone, their inclusion with other wavelengths can enhance overall photochemical efficiency – a phenomenon known as the Emerson enhancement effect [14-15, 22]. The practical implication for citrus nursery production of the interaction between far-red light with the other wavelengths was considered when planning this study

Given the complexity of plant responses to light, a multivariate approach is necessary to disentangle the effects of spectral quality and intensity as well as identify optimal supplemental lighting strategies. In citrus nurseries, where winter light limitations delay graft maturation, LED systems can provide the specific spectra to enhance budbreak as observed in preliminary trials [2]. This study was thus designed to evaluate the effects of supplemental LED lighting, varying in both spectra and intensity, on budbreak and growth of grafted citrus during winter, using response surface methodology to model the interactions among multiple light parameters. The results demonstrated that combinations of blue and white light promote vegetative budbreak, while red and far-red light can suppress budbreak at higher intensities. Conversely, a full-

spectrum approach (red, blue, white, and far-red at high intensity) enhances scion shoot growth. These findings provide insights into the role of light in citrus nursery production and confirm that a multivariate approach is essential for studying and optimizing supplemental lighting. The overall aim – to determine how combinations of light spectra and intensity affect budbreak and growth was achieved, revealing complex interactions that can inform best practices for citrus propagation under controlled conditions.

Materials and Methods

Plant material and growth conditions

Citrus rootstock seedlings were cultivated using two varieties that exhibit a high frequency of nucellar polyembryony and apomictic seedlings: Carrizo (*Citrus sinensis* × *Poncirus trifoliata*) and Rich 16-6 (*P. trifoliata*). Individual seeds were planted in cone-shaped containers measuring 3.8 cm in diameter and 21 cm in height (Cone-tainers; Stuewe and Sons, Tangent, OR, USA). These containers were filled with a soilless growing medium (Pro Mix BX; Premier Horticulture, Inc., Quakertown, PA, USA). Seedlings exhibiting atypical characteristics were identified through visual inspection of their morphology and subsequently removed from the study. The remaining true-to-type seedlings were then transplanted into larger 2.54 L pots (Treepots; Stuewe and Sons) using the same soilless growing medium. Prior to budding, the diameter of each rootstock trunk was measured 15 cm above the soil surface. This measurement was taken approximately two weeks before budding, and any plants found to be undersized were excluded from the experiment. To maintain uniformity within treatment groups, the remaining plants were categorized based on their trunk diameter and randomly assigned to different treatments.

On November 21, 2025 six months old seedlings from Carrizo and Rich 16-6 were budded with Washington Navel orange (*C. sinensis*) using the inverted T bud method. Grafted buds were wrapped with bud tape for 3 weeks. One week after unwrapping, the rootstock was trimmed at 65 cm and looped to force bud growth as described [23]. Plants were arranged on the bench with the grafted bud facing south to increase vegetative budbreak and scion shoot growth [24].

Plants were grown in a temperature-controlled greenhouse set at 21 to 27 °C, controlled by heaters, cooling fans, wet pad and circulating fans. Temperature controls were set to run heating

when temperature dropped below 21 °C, and run cooling when temperature rose above 27 °C. Plants were irrigated to run-off every other week with water-soluble fertilizer, and as needed with water between fertilization treatments. The fertilizer used was 20N-10P-20K (Peters Professional, The Scotts Company, Marysville, OH) at a rate of 400 mg N/L. Insecticides and miticides were applied as needed.

LED light treatments

Plants received sunlight during the day and supplemental LED light treatments were provided during early morning to extend the daylength to 16 hours. The light sources used were light-emitting diode (LED) Elixia 600W (Heliospectra, Göteborg, Sweden). The fixture had four LED types – 1) Red – peak at 660 nm; 2) Blue – peak at 450 nm; 3) White 5700K – peak at 446 nm, 534 nm, and 625 nm; and 4) Far-red – peak at 735 nm. The intensity of each LED light type was varied from 0 to 1,000, the maximum intensity for each wavelength of the fixture. Heliospectra LED lights use a relative, unitless scale for intensity control in their software.

Light fixtures were placed 110 cm above the bud insertion for uniformity of photosynthetic photon flux density (PPFD) distribution among the plants under each light treatment. The timing on the light fixtures was adjusted according to the natural daylength change over the 12-week period of the experiment: every time there was a 15-minute change in the length of natural daylight, the time of the supplemental lighting was adjusted to maintain the 16-hour daylength. Light energy and the average PPFD under each LED light was measured using a Spectrometer PG100N (UPRtek, Zhunan, Taiwan) for each treatment at 110 cm below the light, at bud insertion level, and at night on a moonless night.

Air temperature

A HOBO data logger, model MX2203 (Onset Computer Corporation, MA, USA), was installed among the plants in each treatment and the air temperature was recorded every 10 minutes from the date of budding to 12 weeks-after-budding (wab).

Leaf surface temperature

Measurements of the leaf surface temperature were taken at 12 wab with a hand-held infrared radiometer MI-210 (Apogee Instruments, Logan, UT, USA). The leaf temperature was measured at night, on the first fully expanded leaf from the scion growth on seven different plants under the light in each treatment.

Daily light integral (DLI)

Photosynthetic active radiation (PAR) is the wavelengths of light within the visible range of 400 to 700 nanometers (nm) which drive photosynthesis. The PPFD is the amount of PAR that is hitting the plant in a specific location and time. PPFD was monitored for each treatment using analog full-spectrum quantum sensors SQ-500 (Apogee Instruments, Logan, UT, USA). Each sensor was attached to a data logger and PPFD was measured every 150 seconds and averaged every 900 seconds. DLI was calculated for each treatment using the formula:

$$DLI = \frac{\Sigma \text{PPFD x } 900}{1{,}000{,}000}$$

Responses measured

Eight responses were measured as follows:

1) Budbreak % – Percentage of vegetative buds that had formed shoots by 12 wab (scion bud initiation: shoot tip of bud at least 2 mm long).

2) Bud shoot growth – Length (mm) of bud shoots at 12 wab.
3) Scion diameter – Diameter (mm) of the bud shoot 5 cm above the graft union 12 wab.
4) Rootstock diameter – Rootstock stem diameters (mm) were measured at 5 cm below the graft union at 12 wab .
5) Internode length – Scion length was measured up to the last fully expanded leaf and divided by the number of nodes for average of internode length at 12 wab. Measured as mm.
6) Leaf area – Total leaf area of the bud shoot leaves (cm^2) at 12 wab. Only fully expanded leaves were used for leaf area measurement. The total leaf area was measured using a LI-3100C leaf area meter (LI-COR Biosciences, Lincoln, NE, USA).
7) Scion dry weight – The bud shoot was excised at 12 wab and dried in an oven at 70°C to a constant weight (g).
8) Chlorophyll index – Measured the first fully expanded leaf with a SPAD-502 portable chlorophyll meter (Minolta, Spectrum Technologies, Inc., Plainfield, IL, USA). Seven leaves from the 'Washington' Navel scion from each rootstock and light treatment were measured and averaged at 12 wab.

Experimental design and analyses

Response surface methodology (RSM) was used to model the interactions between four LED light spectra (red, blue, white, far-red) and their intensities while also identifying optimal combinations for various horticultural growth measures. RSM enables for the simultaneous analysis of linear, quadratic, and interaction effects – necessary for understanding and optimizing responses like budbreak and shoot elongation. Unlike single-factor experiments or basic factorial

designs, RSM provides greater statistical power to detect nonlinear patterns and synergistic effects, and in a more efficient approach.

The effects of supplemental LED lighting on budbreak and plant growth were evaluated in two citrus rootstocks, Carrizo and Rich 16-6, using a four-factor D-optimal response surface design (Tables 1 and 2). Experimental factors were the “intensity units” of four types of LED lights - blue, red, white, and far-red - with each power input varying from 0 to 1,000 “intensity units”. The spectral power distribution (SPD) profile was generated for each design run (S1-S3 Figs). The design allowed for estimation of main effects, two-factor interactions, and quadratic effects for each of the four LED light types. The core experimental design consisted of 29 runs, including 15 model points, 5 lack-of-fit points, and 5 replicate points for pure error estimation. Four additional points of interest (Runs #3, #7, #18, and #27) were added and treated as lack-of-fit points in the analyses.

Table 1. The four LED light factors and their ranges to construct the experimental design.

Factors	LED (peak wavelength, nm)	Range (intensity units)
A	Red (660)	0 – 1,000
B	Blue (450)	0 – 1,000
C	White (446, 534, 625)	0 – 1,000
D	Far-red (735)	0 – 1,000

Table 2. Four-factor D-optimal quadratic response surface design matrix. PPFD = Photosynthetic Photon Flux Density – a measure of how many photons land in a specific area that the plant can use.

Run	Intensity Units				PPFD μmol m^{-2} s^{-1}
	Red	Blue	White	Far-red	
1	500	750	750	250	426
2	0	500	0	1000	51
3	1000	0	0	0	352
4	1000	1000	500	1000	560
5	1000	0	0	1000	360
6	1000	1000	0	500	426
7	0	0	0	0	0
8	1000	500	1000	1000	628
9	0	1000	1000	1000	320
10	1000	1000	500	1000	554
11	0	1000	1000	1000	320
12	500	1000	0	1000	263
13	1000	1000	1000	0	664
14	0	500	1000	0	282
15	0	500	1000	0	282
16	1000	0	0	1000	360
17	500	250	750	750	392
18	1000	1000	1000	1000	671
19	1000	0	1000	0	588
20	0	0	0	0	0
21	1000	0	1000	0	588
22	1000	1000	0	0	430
23	0	1000	0	0	81
24	0	0	0	1000	6
25	0	0	1000	1000	244
26	500	250	250	250	275
27	250	750	250	500	226
28	750	500	250	750	387
29	1000	500	0	0	398

For each lamp, 96 plants (48 Carrizo and 48 Rich 16-6) were grown. The area under each lamp was divided into four quadrants, with each quadrant containing 24 plants (12 of each rootstock). This arrangement resulted in each lamp illuminating 48 Washington Navel orange plants budded on Carrizo and 48 budded on Rich 16-6 rootstock. In total, 2,784 plants were used in the experiment. For statistical analysis, the response for each treatment design point and rootstock was calculated by averaging the responses of the 12 plants in each quadrant. Analysis of variance (ANOVA) models were constructed using forward selection based on Akaike's Information Criterion (AICc) [25] to select the highest order polynomial model where additional terms were significant at the 0.05 significance level. Model adequacy was evaluated using multiple methods to ensure reliable statistical analyses: Box-Cox analysis [26] was applied to assess the need for data transformation, with transformations performed prior to ANOVA when necessary; the assumption of constant variance was verified through a plot of internally studentized residuals versus predicted responses; adequate precision, a signal-to-noise ratio, was calculated to confirm a value exceeding the recommended threshold of 4 [27], ensuring sufficient model discrimination; a lack-of-fit (LOF) test was conducted to validate that the model captured all systematic variation in the data; Cook's distance analysis identified potential high-leverage outliers influencing model parameters; and three R^2 metrics (R^2, adjusted R^2, and predicted R^2) were analyzed collectively to evaluate model stability, predictive capability, and alignment between experimental and fitted data.

Results

Temperature

The air temperature inside the greenhouse varied from 21 to 27°C, the minimum temperature average ranged from 20 to 25 °C and the maximum temperature average ranged from 23 to 34°C.

Daily light integral (DLI)

The PPFD from daylight increased throughout the experiment and consequently, the average of DLI increased. The total average of DLI under the no supplemental light treatment was 5 $mol \cdot m^{-2} \cdot day^{-1}$. The highest DLI was under treatment 13, which had red + blue + white light at the highest intensity. The amplitude of DLI among the treatments over the twelve weeks varied from 8 to 11 $mol \cdot m^{-2} \cdot day^{-1}$.

Vegetative and physiological measurements

The ANOVA results summarizing the eight plant response variables measured on the Carrizo and Rich 16-6 rootstocks are presented (Tables 3 and 4), with the corresponding datasets provided (S1 File). For each response a description is provided of the range of the data observed, the p-value of the model, the result of the Box Cox analysis and requirements for data transformation, the three R^2 values as general measure of model quality, the significant terms in each model, and the model's settings that optimized a defined maximized response.

Table 3. ANOVA model terms, p-values (Prob. > F), lack-of-fit, R^2, and adequate precision statistics for the effect of light on % budbreak, shoot growth, and scion and rootstock dimeters. Model equations in coded and actual form are provided (S2 File)

	Budbreak (%)		Scion shoot growth (mm)		Scion shoot diameter (mm)		Rootstock diameter (mm)	
**Source**	**Carrizo**	**Rich 16-6**	**Carrizo**	**Rich 16-6**	**Carrizo**	**Rich 16-6**	**Carrizo**	**Rich 16-6**
Model	0.0005	0.0001	3.41e-06	0.0016	6.029e-05	0.0002	3.49e-13	0.0001
A – Red	0.0025	0.6730	0.0043	0.1870	0.0089	0.0010	2.53e-05	8.29e-05
B – Blue	0.0155	0.0264	0.0263	0.4185	0.15355	0.8934	0.0001	-
C – White	-	0.0018	0.0075	0.0640	0.0316	0.9117	1.17e-07	0.0448
D – Far-red	0.6818	0.5092	0.5057	0.4990	0.7755	-	0.0145	0.3818
AB – Red x Blue	-	-	-	-	-	0.0996	0.1138	-
AC – Red x White	-	-	-	-	-	0.1527	-	-
AD – Red x Far-red	0.0160	0.0036	0.0478	0.0280	0.0369	-	-	-
BC – Blue x White	-	-	0.0012	-	0.0062	-	0.1182	-
BD – Blue x Far-red	-	-	0.1139	-	-	-	-	-
CD – White x Far-red	-	-	-	-	-	-	-	0.0603
A^2 – Red^2	0.0613	-	-	-	-	-	0.0333	-
B^2 – $Blue^2$	-	-	-	0.0042	-	0.0310	-	-
C^2 – $White^2$	-	-	-	-	-	-	-	-
D^2 – $Far\text{-}red^2$	-	-	-	-	-	-	-	-
Lack of Fit	0.0166	0.6336	0.0004	0.4902	0.0074	0.8313	0.1182	0.5650
R^2	0.1733	0.1958	0.2809	0.1748	0.2248	0.2198	0.4723	0.1947
R^2 adj	0.1371	0.16055	0.2360	0.1293	0.1833	0.1748	0.4393	0.1643
R^2 pred	0.0823	0.1083	0.1871	0.0638	0.1350	0.1126	0.4019	0.1100
Adequate precision	7.6325	8.4574	8.1371	7.8005	8.4508	7.0246	13.8638	8.1618
Transformation	no	square root	no	no	no	no	no	no
Model type	reduced quadratic	reduced 2FI	reduced 2FI	reduced quadratic	reduced 2FI	reduced quadratic	reduced quadratic	reduced 2FI

Table 4. ANOVA model terms, p-values (Prob. > F), lack-of-fit, R^2, and adequate precision statistics for the effect of light on internode length, leaf area, scion biomass, and chlorophyll index. Model equations in coded and actual form are provided (S2 File)

	**Internode length (mm)**		**Leaf area (cm^2)**		**Scion dry weight (g)**		**Chlorophyll index**	
**Source**	**Carrizo**	**Rich 16-6**	**Carrizo**	**Rich 16-6**	**Carrizo**	**Rich 16-6**	**Carrizo**	**Rich 16-6**
Model	0.0002	0.0034	3.09e-09	0.0002	5.23e-09	2.43e-07	2.144e-31	1.34e-17
A – Red	0.0462	0.0350	0.0070	0.0262	2.10e-05	3.55e-05	0.0193	1.49e-05
B – Blue	0.0429	0.9080	0.0026	0.9363	0.0109	0.9744	7.97e-13	5.10e-14
C – White	0.7427	0.46448	0.0020	0.0276	0.0028	0.0051	3.96e-15	0.0273
D – Far-red	0.2988	0.2289	0.9482	0.9280	0.9753	0.0407	2.05e-22	0.0001
AB – Red x Blue	-	-	-	-	-	-	0.0373	-
AC – Red x White	-	0.0142	-	-	-	0.0946	-	-
AD – Red x Far-red	0.0786	-	0.0018	0.0187	0.0370	-	2.56e-05	-
BC – Blue x White	0.0024	0.1719	0.0001	0.1181	0.0009	-	-	-
BD – Blue x Far-red	-	0.1180	-	-	-	-	0.0657	0.0236
CD – White x Far-red	-	-	0.0689	-	-	-	0.0002	0.0191
A^2 – Red^2	-	-	-	-	-	-	-	0.0009
B^2 – $Blue^2$	-	0.0418	-	0.0026	-	0.0308	-	0.0208
C^2 – $White^2$	-	-	-	-	-	-	7.28e-10	-
D^2 – $Far\text{-}red^2$	-	-	-	-	-	-	-	-
Lack of Fit	0.0056	0.2727	0.0002	0.8579	0.0125	0.2641	5.28e-21	1.14e-13
R^2	0.2068	0.1969	0.3730	0.2369	0.3515	0.3234	0.7726	0.5758
R^2 adj	0.1646	0.1339	0.3339	0.1850	0.3171	0.2844	0.7539	0.5453
R^2 pred	0.1122	0.0392	0.2810	0.1150	0.2729	0.2297	0.7353	0.5105
Adequate precision	8.4989	6.7292	11.6725	9.1737	11.7080	9.0753	24.1321	15.8894
Transformation	$\lambda = 1.49$	no	square root	square root	square root	no	no	no
Model type	reduced 2FI	reduced quadratic	reduced 2FI	reduced quadratic	reduced 2FI	reduced quadratic	reduced quadratic	reduced quadratic

Percentage Budbreak

Bud survival on Carrizo and Rich 16-6 was 98.1% and 96.6% on average. Of the surviving buds, the percentage budbreak ranged from 8 to 92% for Carrizo and 0 to 58% for Rich 16-6, indicating light affected percentage budbreak. A reduced quadratic polynomial model was selected for Carrizo ($p < 0.001$) and Rich 16-6 ($p < 0.001$). Data were transformed for Rich 16-6 per violation of the ANOVA assumption of normally distributed residuals as detected via Box-Cox analysis. A summary of the ANOVA, lack-of-fit test, three R^2 statistics, and adequate precision statistic for % budbreak is presented (Table 3). The lack-of-fit (LOF) test was significant for Carrizo ($p = 0.0166$). The significant LOF was interpreted to be caused by small error estimates rather than an indication that additional variation could be removed with a better model. For both rootstocks R^2, R^2_{adj} and R^2_{pred} statistics were tightly clustered, R^2 - R^2_{adj} and R^2_{adj} - $R^2_{pred} < 0.2$, and adequate precision was > 4 indicating adequate models for prediction. The ANOVA for Carrizo contained 3 significant terms ($p < 0.05$) – Red, Blue, and Red x Far-red. The ANOVA for Rich 16-6 contained three significant terms, the main effects Blue and White, and Red x Far-red. For Carrizo, the single largest effect was Red. Red and Red x Far-red reduced predicted % budbreak (Fig 1A). For Rich 16-6, the largest effects were White and Red x Far-red; white increased % budbreak and Red and Far-red reduced % budbreak, respectively (Fig 3A).

Scion shoot diameter

The diameter of shoots growing from the grafted buds ranged from 1.6 – 5.1 mm for Carrizo and 0.8 – 4.1 mm for Rich 16-6); the wide range indicated that light affected the diameter of the bud shoots on both rootstocks. The overall models were significant for Carrizo ($p < 0.001$) and Rich 16-6 ($p < 0.001$), indicating significant factor effects on bud shoot diameter. A summary of the ANOVA, lack-of-fit test, three R^2 statistics, and adequate precision statistic for bud shoot

diameter is presented (Table 3). A reduced 2-factor interaction (2FI) polynomial model was selected for Carrizo and a reduced quadratic model for Rich 16-6. Per the Box-Cox analyses no data transformations were required. The lack-of-fit (LOF) tests were significant for Carrizo ($p=0.0074$) and not significant for Rich 16-6 ($p = 0.8313$). The significant LOF for Carrizo was interpreted to be caused by small error estimates. The not significant LOF for Rich 16-6 indicated that additional variation in the residuals could not be removed with a better model. For Carrizo and Rich 16-6 the three R^2 statistics were clustered with a difference less than 0.4 between R^2 and $R^2_{predicted}$, and the adequate precision statistics were greater than 4 as recommended for prediction. The ANOVA for Carrizo contained 4 significant terms ($p < 0.05$) – Red, White, Red x Far-red, and Blue x White. The ANOVA for Rich 16-6 contained 2 significant terms ($p<0.05$) – Red and Blue^2. The models for both rootstocks indicate that maximum scion shoot diameter occurs at the maximum LED factor settings (Fig 1, Fig 3) which resulted in 18% and 41% larger diameters for Carrizo and Rich 16-6, respectively.

Rootstock diameter

The diameter of the rootstocks ranged from 6.1 – 8.7 mm for Carrizo and 3.8 – 6.1 mm for Rich 16-6; the wide range indicated that light affected the diameter of the rootstocks. The overall models were significant for Carrizo ($p < 0.001$) and Rich 16-6 ($p < 0.001$), indicating significant factor effects on rootstock diameter. A summary of the ANOVA, lack-of-fit test, three R^2 statistics, and adequate precision statistic for rootstock diameter is presented (Table 3). A reduced quadratic model was selected for Carrizo and a reduced 2FI model for Rich 16-6. Per the Box-Cox analysis no data transformations were required. The lack-of-fit (LOF) tests were not significant for Carrizo and Rich 16-6 and indicated that additional variation in the residuals could not be removed with a better model. For Carrizo and Rich 16-6 the three R^2 statistics were

clustered with a difference less than 0.4 between R^2 and $R^2_{predicted}$, and the adequate precision statistics were greater than 4 as recommended for prediction. The ANOVA for Carrizo contained 5 significant terms ($p < 0.05$) – Red, Blue, White, Far-red, and Red^2. The greatest effects were White ($p < 0.001$) and Red ($p < 0.001$) that predicted an increased diameter of 6%. The ANOVA for Rich 16-6 contained 2 significant terms ($p<0.05$) – Red and White. The models show that the largest rootstock diameter occurs at low intensity Far-red and high intensity Red, White, and Blue (Fig 1, Fig 3) with an increase in predicted diameter of 12%.

Internode length

The internode lengths of bud shoots ranged from 5.8 – 24.2 mm for Carrizo and 3.3 – 21.5 mm for Rich 16-6; the wide range indicated that light affected internode length. The overall models were significant for Carrizo ($p < 0.001$) and Rich 16-6 ($p = 0.0034$), indicating significant factor effects on internode length. A summary of the ANOVA, lack-of-fit test, three R^2 statistics, and adequate precision statistic for rootstock diameter is presented (Table 4). A reduced 2FI model was selected for Carrizo and a reduced quadratic model for Rich 16-6. Data was transformed for Carrizo per violation of the ANOVA assumption of normally distributed residuals as detected via a Box-Cox analysis. For Rich 16-6 the Box-Cox analysis indicated that no data transformation was required. The lack-of-fit (LOF) test for Carrizo was significant and was interpreted to be caused by small error estimates rather than an indication that additional variation could be removed with a better model. The LOF for Rich 16-6 was not significant and indicated that additional variation in the residuals could not be removed with a better model. For Carrizo and Rich 16-6 the three R^2 statistics were clustered with a difference less than 0.4 between R^2 and $R^2_{predicted}$, and the adequate precision statistics were greater than 4 as recommended for prediction. The ANOVA for Carrizo contained 3 significant terms ($p < 0.05$) – Red, Blue, and

Blue x White. The model shows that the longest internodes were observed at high levels of Blue and White (Fig 2) with a predicted increase in length of 11% compared to no supplemental light. The ANOVA for Rich 16-6 contained 3 significant terms ($p<0.05$) – Red, Red x White and $Blue^2$. The model showed a positive association between Red, White, and Blue and internode length (Fig 4).

Leaf area

Leaf area of bud shoots ranged from 56 – 1,041 cm^2 for Carrizo and 50 – 478 cm^2 for Rich 16-6; the wide range indicated that light affected leaf area. The overall models were significant for Carrizo ($p < 0.001$) and Rich 16-6 ($p < 0.001$), indicating significant factor effects on leaf area. A summary of the ANOVA, lack-of-fit test, three R^2 statistics, and adequate precision statistic for rootstock diameter is presented (Table 4). A reduced 2FI model was selected for Carrizo and a reduced quadratic model for Rich 16-6. Data were transformed for Carrizo and Rich 16-6 per violation of the ANOVA assumption of normally distributed residuals as detected via Box-Cox analysis. The lack-of-fit (LOF) test for Carrizo was significant and was interpreted to be caused by small error estimates rather than an indication that additional variation could be removed with a better model. The LOF for Rich 16-6 was not significant and indicated that additional variation in the residuals could not be removed with a better model. For Carrizo and Rich 16-6 the three R^2 statistics were clustered with a difference less than 0.4 between R^2 and $R^2_{predicted}$, and the adequate precision statistics were greater than 4 as recommended for prediction. The ANOVA for Carrizo contained 5 significant terms ($p < 0.05$) – Red, Blue, White, Red x Far-red, and Blue x White. The model predicted that the greatest leaf area occurs at high levels Red, Blue and White (Fig 2). The ANOVA for Rich 16-6 contained 4 significant terms ($p<0.05$) – Red, White,

Red x Far-red, and Blue^2. The model predicted that the greatest leaf area occurs at high levels of Red, White, Blue and Far-red (Fig 4).

Scion dry weight

Scion dry weight ranged from 1 – 12 g for Carrizo and 0.83 – 5.3 g for Rich 16-6 (Table of data – supplement); the wide range indicated that light affected scion dry weight. The overall models were significant for Carrizo ($p < 0.001$) and Rich 16-6 ($p < 0.001$), indicating significant factor effects on scion dry weight. A summary of the ANOVA, lack-of-fit test, three R^2 statistics, and adequate precision statistic for rootstock diameter is presented (Table 4). A reduced 2FI model was selected for Carrizo and a reduced quadratic model for Rich 16-6. Data were transformed for Carrizo per violation of the ANOVA assumption of normally distributed residuals as detected via Box-Cox analysis. The lack-of-fit (LOF) test for Carrizo was significant and was interpreted to be caused by small error estimates rather than an indication that additional variation could be removed with a better model. The LOF for Rich 16-6 was not significant and indicated that additional variation in the residuals could not be removed with a better model. For Carrizo and Rich 16-6 the three R^2 statistics were clustered with a difference less than 0.4 between R^2 and $R^2_{predicted}$, and the adequate precision statistics were greater than 4 as recommended for prediction. The ANOVA for Carrizo contained 5 significant terms ($p < 0.05$) – Red, Blue, White, Red x Far-red, and Blue x White. The model shows that the greatest dry weight occurs at high levels of Red, Blue, White, and Far-red (Fig 2). The ANOVA for Rich 16-6 contained 5 significant terms ($p<0.05$) – Red, White, Far-red, Red x White, and Blue^2. The model predicted that the greatest dry weight occurs at high levels of Red, White, and Blue (Fig 4).

Chlorophyll index

The leaf chlorophyll index ranged from 71.6 – 81.6 for Carrizo and 71.5 – 82.8 for Rich 16-6 (Table of data – supplement); the wide range indicated that light affected the leaf chlorophyll index. The overall models were significant for Carrizo ($p < 0.001$) and Rich 16-6 ($p < 0.001$), indicating significant factor effects on the leaf chlorophyll index. A summary of the ANOVA, lack-of-fit test, three R^2 statistics, and adequate precision statistic for rootstock diameter is presented (Table 4). A reduced quadratic model was selected for Carrizo and Rich 16-6. For Carrizo and Rich 16-6 the Box-Cox analyses indicated that no data transformations were required. The lack-of-fit (LOF) tests for Carrizo and Rich 16-6 were significant and were interpreted to be caused by small error estimates rather than an indication that additional variation could be removed with a better model. For Carrizo and Rich 16-6 the three R^2 statistics were clustered with a difference less than 0.4 between R^2 and $R^2_{predicted}$, and the adequate precision statistics were greater than 4 as recommended for prediction. The ANOVA for Carrizo contained 8 significant terms ($p < 0.05$) – Red, Blue, White, Far-red, Red x Blue, Red x Far-red, White x Far-red, and $White^2$. The model predicted that the greatest leaf chlorophyll index measure occurs at high levels of Blue and low levels of Far-red (Fig 2). The ANOVA for Rich 16-6 contained 8 significant terms ($p<0.05$) – Red, Blue, White, Far-red, Blue x Far-red, White x Far-red, Red^2, and $Blue^2$. The model predicted that the greatest leaf chlorophyll index measures occur at high levels of Red, Blue, and White, and low levels of Far-red (Fig 4).

Discussion

This study evaluated the effects of supplemental LED light spectra and intensity on the winter growth of grafted citrus nursery plants, focusing on vegetative budbreak and scion shoot development. The findings demonstrate that both the spectral quality and intensity of supplemental LED lighting significantly influence key physiological and morphological parameters in young citrus trees, with implications for optimizing nursery production during periods of limited natural light.

Effects of Light Spectra on Budbreak and Growth

The results indicate that blue and white light, particularly at higher intensities, promote vegetative budbreak of the scion on both Carrizo and Rich 16-6 rootstocks, while red and far-red light tend to suppress budbreak when applied at higher intensities. These findings are consistent with previous studies in citrus and other species, where blue light has been shown to stimulate budbreak and enhance the differentiation of axillary buds. For example, higher budbreak percentages were observed in citrus under LED lighting with a pronounced blue peak [2], and similar positive effects of blue light have been reported in wheat [28], plum [29], and rose [30, 31]. The present study further supports the role of blue-enriched spectra in breaking dormancy and initiating shoot growth in grafted citrus. The positive effect of white light on budbreak observed here can be attributed to its high proportion of blue wavelengths, as the white LEDs used had a correlated color temperature (CCT) of 5700K, which is known to emit a broad spectrum with a substantial blue component. This aligns with the understanding that commercial white LEDs often utilize blue diodes with phosphor coatings [32], resulting in a spectrum that includes biologically active wavelengths beyond the blue region [33].

In contrast, red light alone reduced budbreak, a result that mirrors findings in other woody species where red light can suppress the formation of axillary buds and maintain apical dominance. The suppressive effect of red and far-red light on budbreak, particularly at higher intensities, highlights the importance of spectral balance in managing developmental responses in citrus propagation.

Synergistic Effects of Combined Spectra on Shoot Growth

While blue and white light were most effective for budbreak, the greatest scion shoot growth was achieved when red, blue, and white light were combined at maximum intensity. The addition of far-red light to this combination further increased shoot elongation and leaf area, although far-red light alone did not enhance growth. This synergistic effect is consistent with the Emerson enhancement effect, where the simultaneous excitation of both photosystem I (PSI) and photosystem II (PSII) by a combination of PAR (400–700 nm) and far-red photons (700–800 nm) increases the quantum yield of photosynthesis. Previous research has shown that far-red light, while inefficient on its own, can enhance photochemical efficiency and leaf-level photosynthetic rates when combined with shorter wavelengths [34-36] (Hogewoning et al., 2012; Murakami, Matsuda, & Fujiwara, 2018; Zhen & van Iersel, 2017). Thus, the Emerson effect's spectral synergy suggests commercial nurseries could add far-red LEDs over existing red/blue arrays to accelerate the shoot elongation. The study also found that far-red light increased leaf area only when combined with red light, and not with other wavelengths, suggesting that specific spectral interactions are necessary to maximize canopy expansion and light interception. This observation aligns with reports in other crops where far-red supplementation promotes leaf expansion and biomass accumulation under controlled environments [12, 14, 15, 37-44].

Chlorophyll Content and Photoprotection

Chlorophyll content was highest under blue and white light and lowest under far-red light, with the chlorophyll index increasing with blue light intensity. These results are in agreement with prior studies showing that blue light is essential for chlorophyll synthesis and prevents etiolation, while red light alone is insufficient [45-49]. The ability of plants to adjust chlorophyll content and the chlorophyll a/b ratio in response to light quality reflects adaptive mechanisms for optimizing photosynthetic efficiency and photoprotection under varying spectral conditions [31, 47, 48, 50-53].

Rootstock-Specific Responses and Light Spectrum Correlations

The study identified both shared and rootstock-specific morphological responses to light spectra in Carrizo and Rich 16-6. While blue and white light enhanced vegetative budbreak in both rootstocks, inhibitory effects from red and far-red light were more pronounced in Carrizo. After bud emergence, all tested spectra positively influenced subsequent bud development, though spectral associations with specific growth traits differed. Once buds exceeded 2 mm, continued growth occurred under all spectral combinations, but trait optimization required wavelength-specific combinations: red light enhanced scion dry weight despite suppressing initial budbreak, far-red light reduced chlorophyll content but synergized with other spectra to increase leaf area, and white light had the strongest correlation with rootstock trunk growth, particularly in Carrizo, where it had the single largest effect. Being broad-spectrum, white light likely optimized photoassimilate allocation. In contrast, far-red light (735 nm) showed the highest correlation with internode elongation, especially in Rich 16-6, where its interaction with red light increased internode length by 18% compared to far-red-deficient spectra, an effect consistent with far-red's known role in phytochrome-mediated shade avoidance responses where the red-to-far-red (R:FR)

light ratio regulates shoot elongation [54]. These results indicate the need for phase-specific spectral management—prioritizing blue/white light for dormancy release, then specific red-far-red ratios for canopy or rootstock development. Rootstock genotype further modulated responses, with Carrizo showing greater sensitivity to far-red during the early growth stage.

Implications and Future Directions

The results of this study have practical implications for commercial citrus nurseries. By optimizing supplemental LED light spectra and intensity, it is possible to enhance budbreak and shoot growth during winter, potentially reducing the duration of a production cycle and improving propagation efficiency. The findings support a multivariate approach to light management, considering both the main effects and interactions among different wavelengths. Future research should explore the underlying physiological mechanisms driving these responses, including hormonal regulation and gene expression associated with light perception and signaling. Additionally, field validation of these findings under commercial nursery conditions would help refine best practices for supplemental lighting. Further investigation into cultivar-specific and rootstock-specific responses to light spectra could enable more targeted management strategies.

Conclusion

This study demonstrated that supplemental LED lighting, with controlled spectra and intensity, can enhance the winter growth of grafted citrus nursery plants. By extending the photoperiod to 16 hours and manipulating the proportions of blue, red, white, and far-red wavelengths, it is possible to influence both vegetative budbreak and scion shoot development in young citrus trees. Blue and white light, particularly at higher intensities, were most effective in promoting budbreak, while the greatest shoot elongation and leaf area were achieved when all four spectra were combined at maximum intensity.

The responses observed were generally consistent across the two rootstocks tested, Carrizo and Rich 16-6, suggesting that these lighting strategies may be broadly applicable within citrus nursery production. These findings support the use of multivariate approaches to optimize supplemental lighting, considering both the main effects and interactions among different wavelengths.

The results provide a practical basis for improving nursery efficiency during periods of limited natural light, potentially reducing production time and enhancing propagation success. However, the study was conducted under specific controlled greenhouse conditions, and further research is warranted to validate these findings under a range of commercial nursery environments and across a wider range of citrus genotypes.

Overall, this work contributes to the understanding of how precise manipulation of light quality and intensity can be used to improve citrus nursery production and provides guidance for the development of best practices in supplemental lighting management.

Conflict of Interest

The authors declare that the research was conducted in the absence of any commercial or financial relationships that could be considered as a potential conflict of interest.

Author contributions

R.B.B.: planning, data collection and formal data analysis; writing - original draft and editing. U.A. and K.D.B.: conceptualization; planning, resources; funding acquisition; supervision; writing – review and editing. R.P.N: experimental design, analysis, and writing. All authors approved the submission.

Funding

This research was conducted at the United States Horticultural Research Laboratory, Ft Pierce, Florida, USA and was funded by the California Citrus Nursery Board (ALB-21-23 and BOW-21-23).

Acknowledgments

We would like to thank Sailindra Patel and Kerry Worton for their help with tree manipulations, data collection, and sample collection and analysis.

References

1. Bisi RB, Bowman KD, Albrecht U. Increasing sweet orange growth in the winter nursery with supplemental light and heating. Horticulturae. 2024 Aug 24;10(9):897.
2. Bowman KD, Albrecht U. Improving winter growth in the citrus nursery with LED and HPS supplemental lighting. HortScience. 2021;56(1):21-7.
3. Brar GS, Spann TM. Vegetative growth and carbohydrate partitioning in citrus nursery trees in response to photoperiod and light interruption. HortScience. 2014;49(8):1005-10.
4. Inoue H. Effects of photoperiod and temperature on growth and floral induction in Satsuma mandarin (Citrus unshiu Marcovitch). Bull Fruit Tree Res Stn Ser E (Okitsu). 1989;12:1-23.
5. Nauer EM, Carpenter JB, Sites WC. Influence of extended daylength and temperature on growth of citrus nursery trees. Proc Int Soc Citric. 1979;1:117-20.
6. Piringer AA, Downs RJ, Borthwick HA. Effects of photoperiod and kind of supplemental light on the growth of three species of citrus and Poncirus trifoliata. Proc Amer Soc Hort Sci. 1961;77:202-10.
7. Young RH. Influence of day length, light intensity and temperature on growth, dormancy and cold hardiness of red-blush grapefruit trees. Proc Amer Soc Hort Sci. 1961;78:174-80.
8. Devlin PF, Yanovsky MJ, Kay SA. Plant photoreceptors. In: Hall JL, Leaver HG, Trewavas GTT, editors. Annual Plant Reviews. Vol. 21. Plant Cell Separation and Adhesion. Oxford: Blackwell Publishing; 2007. p. 1-36.
9. Paradiso R, Proietti S. Light-quality manipulation to control plant growth and photomorphogenesis in horticultural crops. Front Plant Sci. 2022;13:978468.
10. Kelly N, Choe D, Meng Q, Runkle ES. Longer photoperiods with the same daily light integral improve growth of lettuce seedlings under sole-source lighting. HortScience. 2020;55(10):1676-82.
11. Zheng L, Van Labeke MC, Van Der Straeten D. Blue light increases anthocyanin content and modifies the expression of light-sensitive genes in Chinese bayberry (Myrica rubra Sieb. et Zucc.). Hortic Environ Biotechnol. 2019;60:357-67.
12. Park Y, Runkle ES. Far-red radiation promotes growth of seedlings by increasing leaf expansion and whole-plant net assimilation. Environ Exp Bot. 2017;136:41-9.
13. Liu J, van Iersel MW. Far-red light effects on lettuce growth and morphology in indoor production are cultivar specific. Plants (Basel). 2022;11(20):2714.
14. Zhen S, Bugbee B. Far-red photons have equivalent efficiency to traditional photosynthetic photons: Implications for redefining photosynthetically active radiation. Plant Cell Environ. 2020;43(5):1259-72.
15. Zhen S, Bugbee B. Substituting far-red for traditionally defined photosynthetic photons results in equal canopy quantum yield for CO_2 fixation and increased photon capture during long-term studies: Implications for re-defining PAR. Front Plant Sci. 2020;11:581156.
16. Modarelli DA, Arena C, Vitale L, De Micco V. High Light Intensity from Blue-Red LEDs Enhance Photosynthetic Performance and Carotenoid Accumulation in

Micropropagated Olive Plants. Horticulturae. 2022;8(2):114.
17. Takahashi S, Badger MR. Photoprotection in plants: a new light on photosystem II damage. Trends Plant Sci. 2011;16(1):53-60.
18. Karpiński S, Szechyńska-Hebda M, Wituszyńska W, Burdiak P. Light acclimation, retrograde signalling, cell death and immune defences in plants. Plant Cell Environ. 2013;36(4):736-44.
19. Naznin MT, Lefsrud M, Gravel V, Azad MOK. Blue Light added with Red LEDs Enhance Growth Characteristics, Pigments Content, and Antioxidant Capacity in Lettuce, Spinach, Kale, Basil, and Sweet Pepper in a Controlled Environment. Plants (Basel). 2019;8:93.
20. Dougher TAO, Bugbee B. Evidence for yellow light suppression of lettuce growth. Photochem Photobiol. 2001;73(2):208-12.
21. Dierck R, Dhooghe E, Van Huylenbroeck J, Van Der Straeten D, De Keyser E. Light quality regulates plant architecture in different genotypes of Chrysanthemum morifolium Ramat. Sci Hortic. 2017;218:177-86.
22. Emerson R, Cayle T. Effect of wave-length on the distribution of carbon-14 in the early products of photosynthesis. Nature. 1957;179(4553):89-90.
23. Bowman KD. Comparison of Two Citrus Bud-forcing Methods for Rapid Propagation of Scions on New Hybrid Citrumelo Rootstocks. HortScience. 1999;34:142-3.
24. Niedz RP, Bowman KD. Improving citrus bud grafting efficiency. Sci Rep. 2023;13:17807.
25. Akaike HA. New Look at the Statistical Model Identification. IEEE Trans Automat Contr. 1974;19(6):716-23.
26. Box GEP, Cox DR. An analysis of transformations. J R Stat Soc Ser B. 1964;26:211-52.
27. Anderson MJ, Whitcomb PJ. RSM simplified: optimizing processes using response surface methods for design of experiments. New York: Productivity Press; 2005.
28. Barnes C, Bugbee B. Morphological responses of wheat to blue light. J Plant Physiol. 1992;139(3):339-42.
29. Muleo R, Morini S, Casano S. Photoregulation of growth and branching of plum shoots: physiological action of two photosystems. In Vitro Cell Dev Biol Plant. 2001;37(5):609-17.
30. Girault T, Abidi F, Sigogne M, Guillot T, Soubigou-Taconnat L, Pelleschi-Travier S, et al. Sugars are under light control during bud burst in Rosa sp. Plant Cell Environ. 2008;31(10):1445-56.
31. Abidi F, Girault T, Douillet O, Guillemain G, Sintes G, Laffaire M, et al. Blue light effects on rose photosynthesis and photomorphogenesis. Plant Biol. 2013;15(1):67-74.
32. Meng Q, Runkle ES. Blue photons from broad-spectrum LEDs control growth, morphology, and coloration of indoor hydroponic red-leaf lettuce. Plants (Basel). 2023;12(5):1127.
33. Bispo-Jr AG, Saraiva LF, Lima SA, Pires AM, Davolos MR. Recent prospects on phosphor-converted LEDs for lighting, displays, phototherapy, and indoor farming. J Luminescence. 2021;237:118167.
34. Hogewoning SW, Wientjes E, Douwstra P, Trouwborst G, van Ieperen W, Croce R, Harbinson J. Photosynthetic quantum yield dynamics: from photosystems to leaves. Plant Cell. 2012;24(5):1921-35.
35. Murakami K, Matsuda R, Fujiwara K. Mathematical model of photosynthetic electron

transport in C3 plants based on the photosystem stoichiometry and alternative electron flows. Plant Cell Physiol. 2018;59(8):1643-51.
36. Zhen S, van Iersel MW. Far-red light is needed for efficient photochemistry and photosynthesis. J Plant Physiol. 2017;209:115-22.
37. Craver JK, Marek S, Lopez RG. Supplemental far-red radiation during seedling production influences subsequent flowering of long-day annuals. Sci Hortic. 2018;235:431-40.
38. Hernandez R, Spalholz H. Supplemental far-red lighting during seedling production accelerates flowering of long-day annuals. HortScience. 2019;54(11):1929-33.
39. Jin S, Sakamoto K, Higashi K. Far-red light enhances lettuce growth by increasing leaf expansion and biomass accumulation under red and blue light. Front Plant Sci. 2021;12:581156.
40. Kubota C, Kroggel M, Both AJ. Plant lighting basics and applications. University of Arizona Controlled Environment Agriculture Center Extension Publication. 2012 Sep:1-7.
41. Legendre L, van Iersel MW. Far-red light enhances photochemical efficiency, leaf expansion, and shoot biomass accumulation in lettuce. Front Plant Sci. 2021;12:730119.
42. Li Q, Kubota C. Effects of supplemental light quality on growth and phytochemicals of baby leaf lettuce. Environ Exp Bot. 2009,67(1):59-64.
43. Meng Q, Runkle ES. Far-red radiation interacts with light quality and intensity to affect growth and morphology of lettuce and kale. Sci Hortic. 2019;255:45-53.
44. Zou J, Zhang Y, Zhang Y, Bian Z, Fanourakis D, Yang Q, et al. Supplemental far-red light increases yield and leaf expansion of lettuce in a vertical farming system. Front Plant Sci. 2019;10:1665.
45. Tripathy BC, Brown CS. Root-shoot interaction in the greening of wheat seedlings grown under red light. Plant Physiol. 1995;107(2):407-11.
46. Hernández R, Kubota C. Physiological responses of cucumber seedlings under different blue and red photon flux ratios using LEDs. Environ Exp Bot. 2014;98:30-7.
47. Hogewoning SW, Trouwborst G, Maljaars H, Poorter H, van Ieperen W, Harbinson J. Blue light dose–responses of leaf photosynthesis, morphology, and chemical composition of Cucumis sativus grown under different combinations of red and blue light. J Exp Bot. 2010;61(11):3107-17.
48. Matsuda R, Ohashi-Kaneko K, Fujiwara K, Goto E, Kurata K. Photosynthetic characteristics of rice leaves grown under red light with or without supplemental blue light. Plant Cell Physiol. 2007;48(2):258-68.
49. Yorio NC, Goins GD, Kagie HR, Wheeler RM, Sager JC. Improving spinach, radish, and lettuce growth under red light-emitting diodes (LEDs) with blue light supplementation. HortScience. 2001;36(2):380-3.
50. Johkan M, Shoji K, Goto F, Hashida SN, Yoshihara T. Blue light-emitting diode light irradiation of seedlings improves seedling quality and growth after transplanting in red leaf lettuce. HortScience. 2010;45(12):1809-14.
51. Kopsell DA, Sams CE. Increases in shoot tissue pigments, glucosinolates, and mineral elements in sprouting broccoli after exposure to light-emitting diodes during growth. J Am Soc Hortic Sci. 2013;138(1):31-7.
52. Ohashi-Kaneko K, Takase M, Kon N, Fujiwara K, Kurata K. Effect of light quality on growth and vegetable quality in leaf lettuce, spinach and komatsuna. Environ Control

Biol. 2007;45(3):189-98.
53. Wang Y, Folta KM, Zhen S. Contributions of green light to plant growth and development. Am J Bot. 2014;101(5):830-42.
54. Franklin KA, Whitelam GC. Phytochromes and shade-avoidance responses in plants. Ann Bot. 2005;96(2):169-75.

Figures

Figure 1. Contour response surface plots of the effects of red, blue, white, and far-red LED light spectra on A) Budbreak percentage, B) Scion shoot growth, C) Scion shoot diameter, and D) Rootstock diameter for Carrizo rootstock.

Figure 2. Contour response surface plots of the effects of red, blue, white, and far-red LED light spectra on A) Internode length, B) Leaf area, C) Scion dry weight, and D) Chlorophyll index for Carrizo rootstock.

Figure 3. Contour response surface plots of the effects of red, blue, white, and far-red LED light spectra on A) Budbreak percentage, B) Scion shoot growth, C) Scion shoot diameter, and D) Rootstock diameter for Rich 16-6 rootstock.

Figure 4. Contour response surface plots of the effects of red, blue, white, and far-red LED light spectra on A) Internode length, B) Leaf area, C) Scion dry weight, and D) Chlorophyll index for Rich 16-6 rootstock.

Figure 1

Click here to access/download;Figure;Figure 1_8-8-2025_2250 W x 2372 H.tif

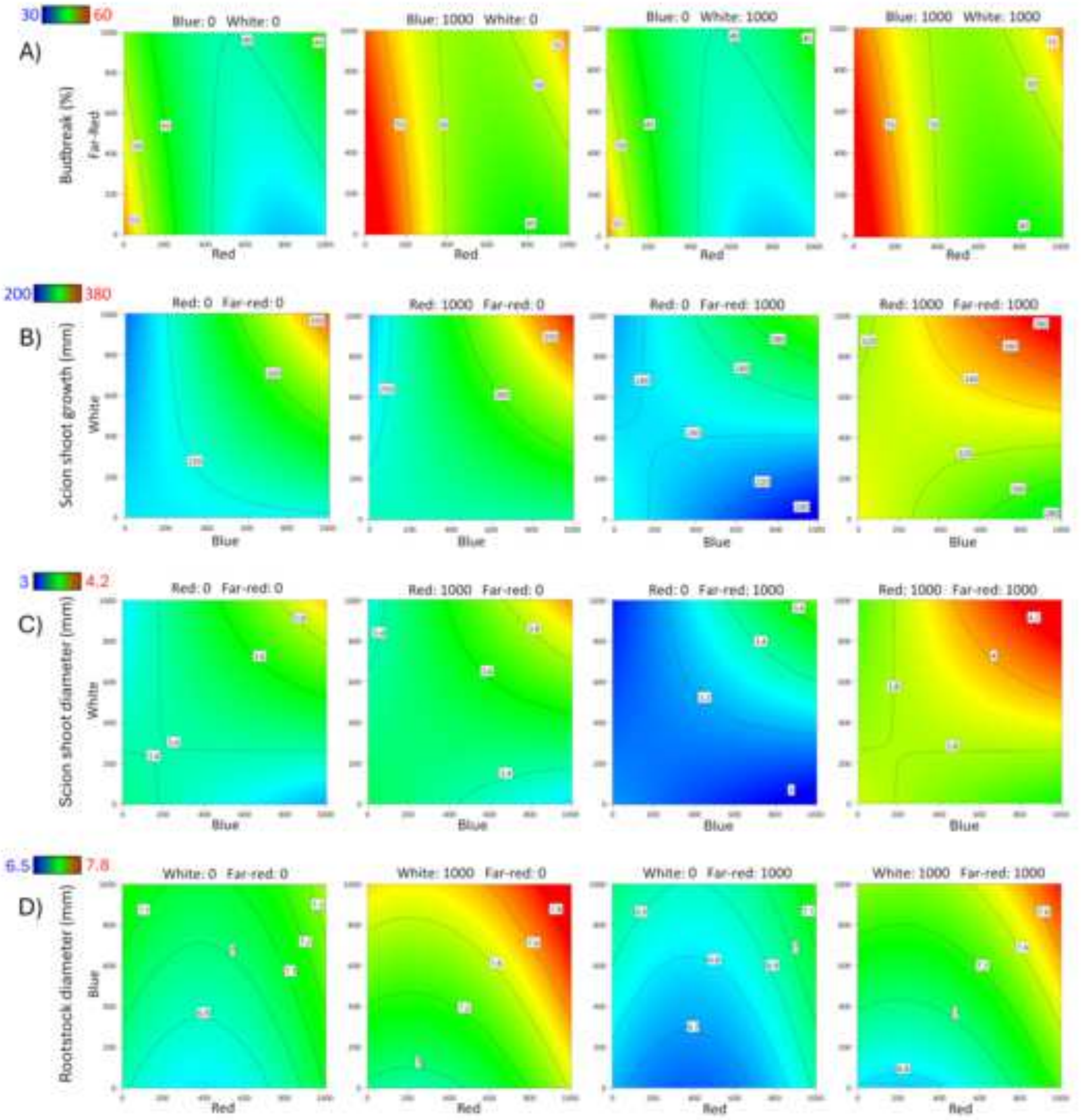

Figure 2

Click here to access/download;Figure;Figure 2_8-8-2025_2250 W x 2337 H.tif

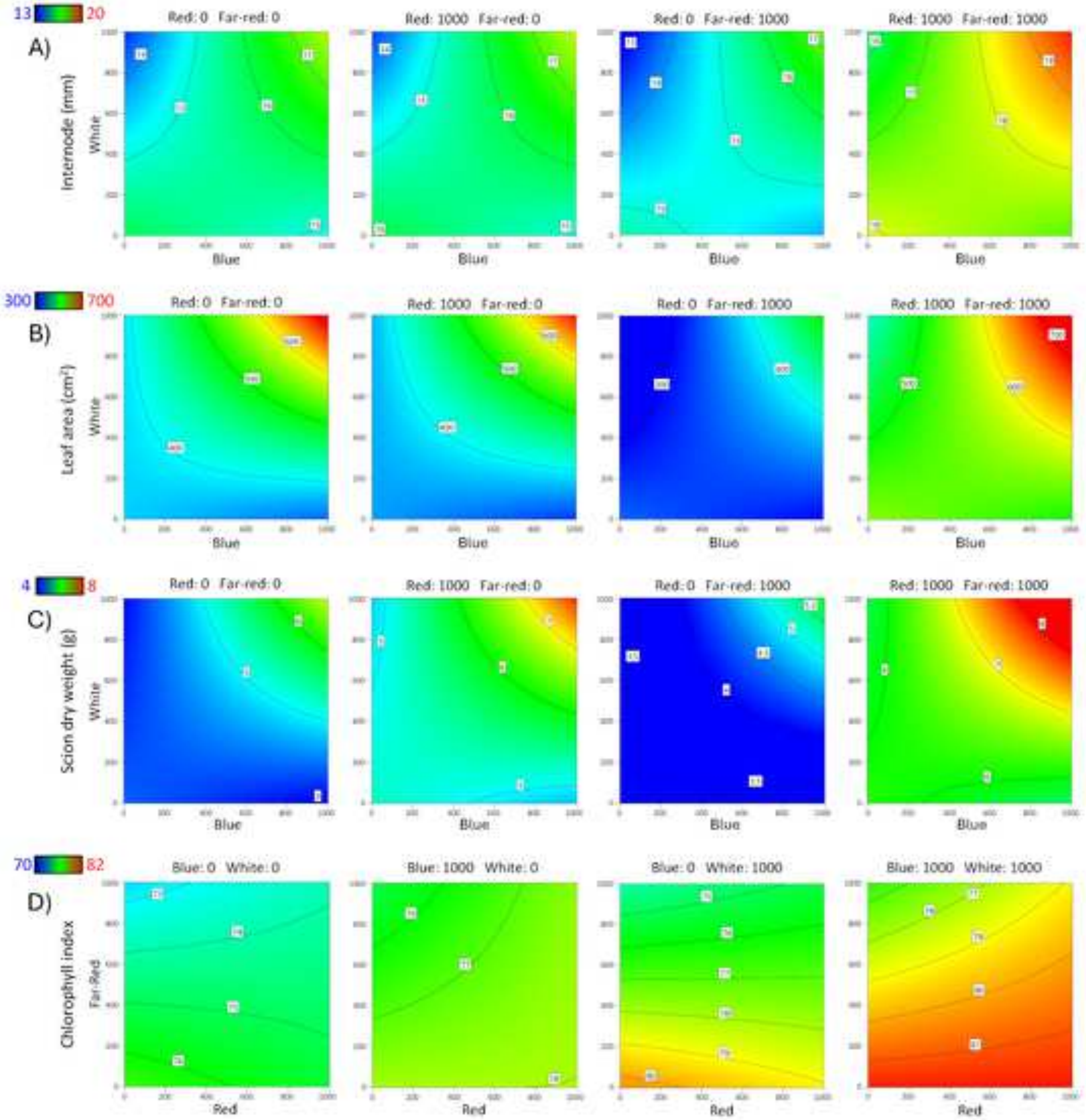

Figure 3

Click here to access/download;Figure;Figure 3_8-8-2025_2250 W x 2347 H.tif

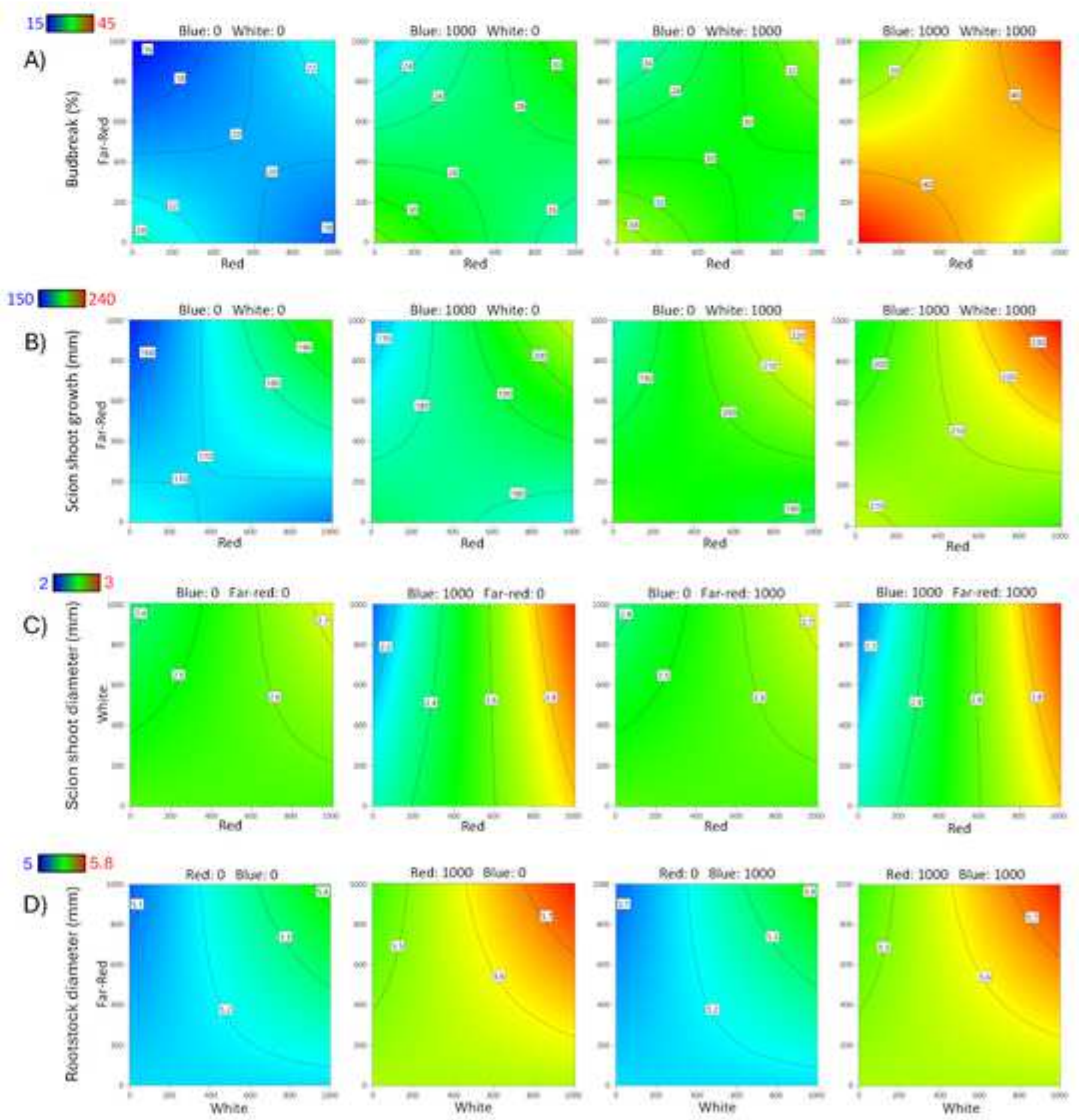

Figure 4

Click here to access/download;Figure;Figure 4_8-8-2025_2250 W x 2324 H.tif

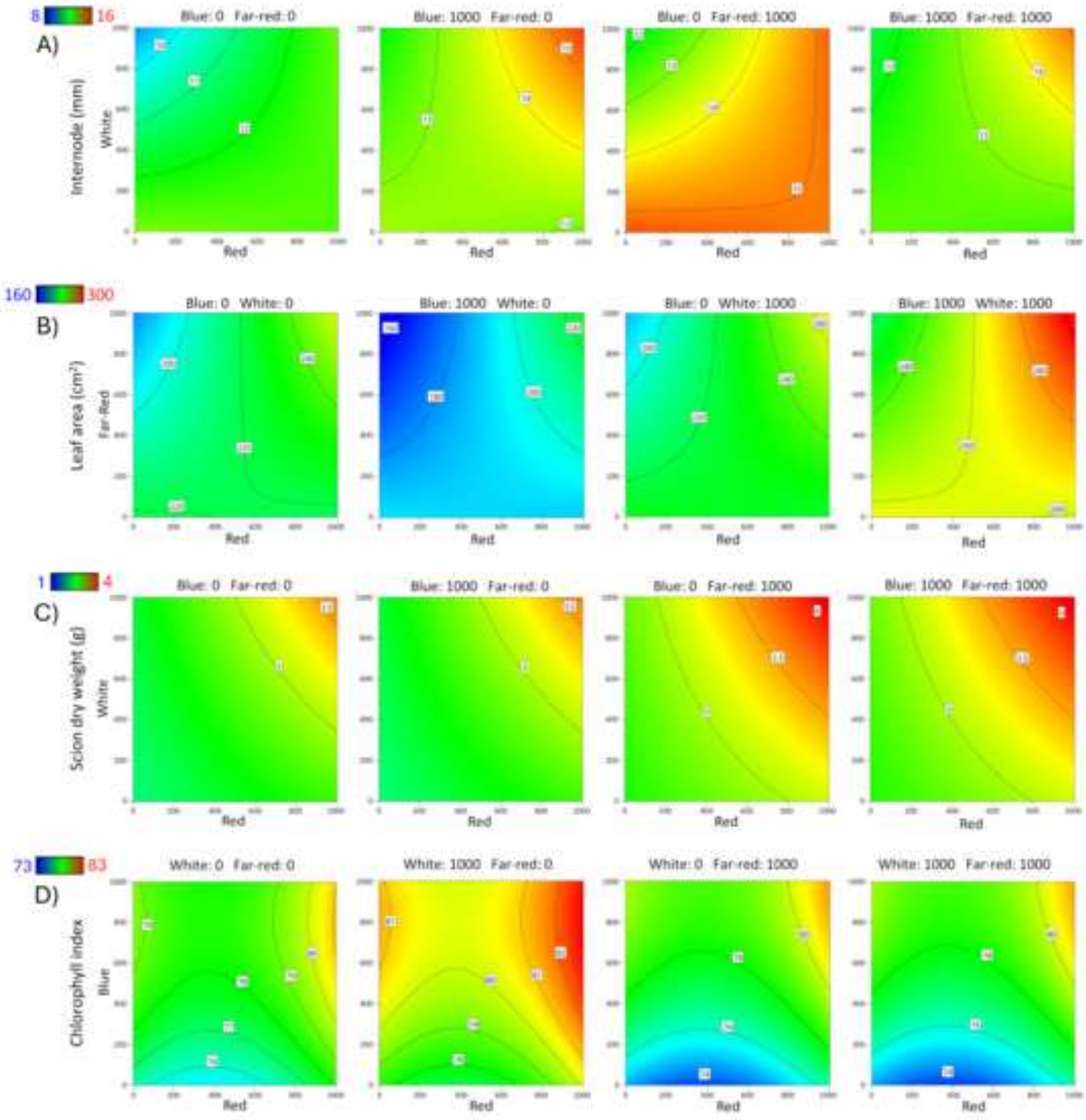

S1 Figure

Click here to access/download
Supporting Information
S1 Fig.tif

S2 Figure

Click here to access/download
Supporting Information
S2 Fig.tif

S3 Figure

Click here to access/download
Supporting Information
S3 Fig.tif

S1 File

Click here to access/download
Supporting Information
S1 File.xlsx

S2 File

Click here to access/download

Supporting Information

S2 File.docx
