# Supplementary material for: Effects of LED light spectra and intensity on winter citrus nursery production
Source: PLoS One. 2026 Apr 30;21(4):e0347764. doi: 10.1371/journal.pone.0347764 (PMC13132180; doi:10.1371/journal.pone.0347764)
Supplement: Attachment — Submitted filename: PONE-D-25-45421-Comments.docx [file pone.0347764.s007.docx]

To Editors

PLOS ONE

**General Assessment**

The manuscript investigates the effects of supplemental LED spectra and intensity on winter growth of grafted citrus nursery plants using a four-factor response surface methodology. The topic is relevant for horticultural lighting optimization, and the dataset appears extensive. However, the manuscript has major issues regarding clarity, structure, statistical interpretation, and figure quality. Substantial revision is needed before the manuscript can be considered for publication.

**Major Comments**

**1. Missing Key Points / Conceptual Gaps**

**No explicit research hypotheses.** The study describes aims but does not articulate testable hypotheses or expected spectral effects.

**Insufficient justification for chosen LED intensities (0–1000 units).** These are “unitless” values, not convertible to PPFD — unclear how they translate to biologically meaningful intensities.

**Lack of physiological mechanism discussion.** While the Discussion includes literature, it does not connect results to plant hormone regulation, phytochrome dynamics, dormancy physiology, or carbon allocation.

**DLI context is missing.** Average DLI of 5–11 mol·m⁻²·day⁻¹ is extremely low; the implications for citrus ecophysiology should be addressed.

**No explanation of seasonal/environmental covariates.** Temperature ranged 21–34°C; this variation could confound spectral effects but is not statistically accounted for.

**Far-red spectrum justification is incomplete.** The manuscript references the Emerson effect but does not evaluate photosynthetic responses or phytochrome photo-equilibria (PPE/R:FR ratio).

**Missing limitations section.** The authors do not acknowledge that the study was conducted in one location, one season, with only two rootstocks.

**2. Issues in Logical Flow**

The Introduction is lengthy and repetitive; the rationale for multivariate RSM appears late and disconnected from the description of winter nursery challenges.

Discussion jumps between topics (budbreak, light effects, synergy) without synthesizing results into clear mechanistic conclusions.

Some causal statements are unsupported or incorrectly implied (e.g., statements connecting far-red to morphological outcomes without citation strength).

The Results are overly statistical, lacking biological interpretation alongside ANOVA outputs.

**3. Research Methodology Concerns**

**Experimental unit unclear.** The study averages 12 plants per quadrant; but the lamp is the experimental unit, not the individual plants. This may artificially inflate sample size and violate independence.

**No replication of lighting treatments.** A single fixture per design point means effects could be due to lamp/bench position, not spectra.

**Quadrant averaging is questionable.** Averaging masks within-treatment variability and ignores plant-to-plant variation.

**RSM adequacy not convincingly demonstrated.**

Many models show low R² (<0.30 in several cases), indicating poor predictive ability.

Significant lack-of-fit in many models suggests model misspecification.

Authors repeatedly state LOF is due to “small error estimates,” an unsubstantiated claim.

**Box-Cox transformation decisions are inconsistently justified.**

**No randomization details.** Pot position, bench orientation, and environmental gradients are not described.

**PPFD and spectra measurements unclear.**

The manuscript does not provide absolute µmol·m⁻²·s⁻¹ per LED channel across treatments.

Lacking calibrated SPD data for each run undermines interpretation.

**4. Results Section – Issues**

Overreliance on ANOVA tables, lacking clear biological synthesis.

Many effect sizes are not quantified (e.g., stating “increased,” “decreased,” without mm or % changes).

Confusion between statistical significance and biological relevance.

Budbreak results interpreted despite poor model R² (~0.17–0.19), making predictions unreliable.

Missing direct comparison between rootstocks, though contrasts are mentioned in Discussion.

**5. Discussion Weaknesses**

Contains excessive literature review instead of critically interpreting results.

Several claims appear overstated relative to the weak model fits.

Does not acknowledge contradictions within results (e.g., red light suppresses budbreak but increases dry weight).

No mention of energy cost or commercial feasibility, despite claiming practical implications.

Discussion does not address R:FR ratio or PPE essential for interpreting far-red responses.

Fails to reconcile budbreak suppression vs. later growth promotion under red light.

**6. Figures and Tables Evaluation**

**Figures**

**Contour plots are low interpretability.**

Colours/gradients unclear; axis labels small; legends missing numerical scales for intensity units.

Hard to compare Carrizo vs. Rich 16-6.

No confidence intervals or standard error surfaces.

**Figures S1–S3 s**hould be incorporated into main text or summarized.

**TIF images appear extremely large and not optimized.**

**Tables**

Tables 3–4 are overloaded, extremely dense, difficult to interpret.

Many P values are overly precise (e.g., 3.09e-09) unnecessary for biological interpretation.

Model type (reduced quadratic, 2FI) is listed but not explained anywhere.

Missing summary table linking optimal spectral combinations for each trait.

**7. Grammar, Style, and Clarity Issues**

Frequent grammatical errors and run-on sentences.

Inconsistent tenses between sections.

Repetition of similar statements across abstract, introduction, and discussion.

Misuse of scientific terminology (e.g., “intensity units” presented as if equivalent to PPFD).

Some sentences are ambiguous or unscientific, (e.g.,
The significant LOF was interpreted to be caused by small error estimates… unclear).

Citations occasionally embedded within sentences without logical connection.
